# Impact of foetal growth restriction and being born small for gestational age on newborn echo- and electrocardiographic measurements—a Copenhagen baby heart study

**DOI:** 10.1093/ehjopen/oeaf177

**Published:** 2026-01-07

**Authors:** Emil H Nørskov, Signe L Skjellerup, Johan E Navne, Raheel A Raja, Maria M Pærregaard, Anne-Sophie Sillesen, Anna A Raja, Alex H Christensen, Kasper K Iversen, Henning Bundgaard, Heather A Boyd, Dorthe L Jeppesen, R Ottilia B Vøgg

**Affiliations:** Department of Cardiology, Herlev-Gentofte Hospital, Borgmester Ib Juuls Vej 1, 2730 Herlev, Denmark; Department of Internal Medicine, Amager-Hvidovre Hospital, Copenhagen University Hospital, Kettegård Alle 36, 2650 Hvidovre, Denmark; Department of Intensive Care, Rigshospitalet, Copenhagen University Hospital, Blegdamsvej 9, 2100 Copenhagen, Denmark; Department of Paediatrics and Adolescent Medicine, Rigshospitalet, Copenhagen University Hospital, Blegdamsvej 9, 2100 Copenhagen, Denmark; Department of Cardiology, Herlev-Gentofte Hospital, Borgmester Ib Juuls Vej 1, 2730 Herlev, Denmark; Department of Cardiology, Rigshospitalet, Copenhagen University Hospital, Blegsvej 9, 2100 Copenhagen, Denmark; Department of Cardiology, Rigshospitalet, Copenhagen University Hospital, Blegsvej 9, 2100 Copenhagen, Denmark; Department of Clinical Medicine, University of Copenhagen, Blegdamsvej 3B, 2200 Copenhagen, Denmark; Department of Cardiology, Herlev-Gentofte Hospital, Borgmester Ib Juuls Vej 1, 2730 Herlev, Denmark; Department of Cardiology, Rigshospitalet, Copenhagen University Hospital, Blegsvej 9, 2100 Copenhagen, Denmark; Department of Clinical Medicine, University of Copenhagen, Blegdamsvej 3B, 2200 Copenhagen, Denmark; Department of Cardiology, Herlev-Gentofte Hospital, Borgmester Ib Juuls Vej 1, 2730 Herlev, Denmark; Department of Clinical Medicine, University of Copenhagen, Blegdamsvej 3B, 2200 Copenhagen, Denmark; Department of Cardiology, Rigshospitalet, Copenhagen University Hospital, Blegsvej 9, 2100 Copenhagen, Denmark; Department of Clinical Medicine, University of Copenhagen, Blegdamsvej 3B, 2200 Copenhagen, Denmark; Department of Epidemiology Research, Statens Serum Institut, Artillerivej 5, 2300 Copenhagen, Denmark; Department of Clinical Medicine, University of Copenhagen, Blegdamsvej 3B, 2200 Copenhagen, Denmark; Department of Paediatrics, Amager-Hvidovre Hospital, Copenhagen University Hospital, Kettegård Alle 36, 2650 Hvidovre, Denmark; Department of Cardiology, Herlev-Gentofte Hospital, Borgmester Ib Juuls Vej 1, 2730 Herlev, Denmark

**Keywords:** Foetal growth restriction, Small for gestational age, Intrauterine growth restriction, Echocardiography, Electrocardiography, Newborn

## Abstract

**Aims:**

Foetal growth restriction (FGR) and being born small for gestational age (SGA) have been linked to later cardiovascular disease. We examined the impact of FGR and SGA on neonatal echo- and electrocardiographic measurements in the Copenhagen Baby Heart Study.

**Methods and results:**

The study included 26 175 newborns. Infants with FGR (*n* = 1020) and SGA (*n* = 2328) were compared to infants born appropriate for gestational age using linear regression with adjustment for body size, age at examination, sex, and gestational age at birth. FGR and SGA were associated with reduced left ventricular (LV) internal diameters in end-systole [adjusted mean difference (aMD) −0.09 mm, 95% confidence interval (CI) −0.18,0.01, and −0.12 mm, 95% CI −0.19, −0.06, respectively] and end-diastole (aMD −0.13 mm, 95% CI −0.24, −0.02, and −0.15 mm, 95% CI −0.22, −0.07, respectively). Exposed newborns also had thinner LV walls [interventricular septum, end-diastole: FGR −0.07 mm (95% CI: −0.11, −0.03), SGA −0.03 mm (95% CI −0.06, −0.01); LV posterior wall, end-diastole: FGR −0.05 mm (95% CI −0.09, −0.01), SGA −0.04 mm (95% CI −0.07, −0.01)]. Both groups exhibited changes in *trans*-mitral flow. FGR was associated with reduced right ventricular function (TAPSE aMD −0.20, 95% CI −0.31, −0.09), whereas SGA was associated with reduced LV end-diastolic (aMD −0.16, 95% CI −0.28, −0.04) and end-systolic volumes (aMD −0.08, 95% CI −0.13, −0.02) and alterations in electrocardiogram precordial leads. Both groups demonstrated reduced uncorrected QT intervals.

**Conclusion:**

Newborns with FGR or SGA exhibited noteworthy cardiac changes, even after adjustment for body size. Follow-up studies are needed to determine the clinical significance of these findings for the later cardiovascular health.

**Key Question:**

Is inadequate foetal growth associated with subclinical changes to the infant heart that can be observed in the newborn using echo- and electrocardiography?

**Key finding:**

Foetal growth restriction and being born small for gestational age were associated with reductions in left ventricular thickness and diameter, changes in diastolic function, and alterations in electrocardiographic parameters. Growth-restricted newborns also had altered right ventricular function.

**Take-home message:**

Foetal growth restriction and being born small for gestational age are associated with alterations in cardiac structure and function, even after adjustment for body size and age. Follow-up examinations of these children should be considered.

## Graphical Abstract

**Graphical Abstract:**
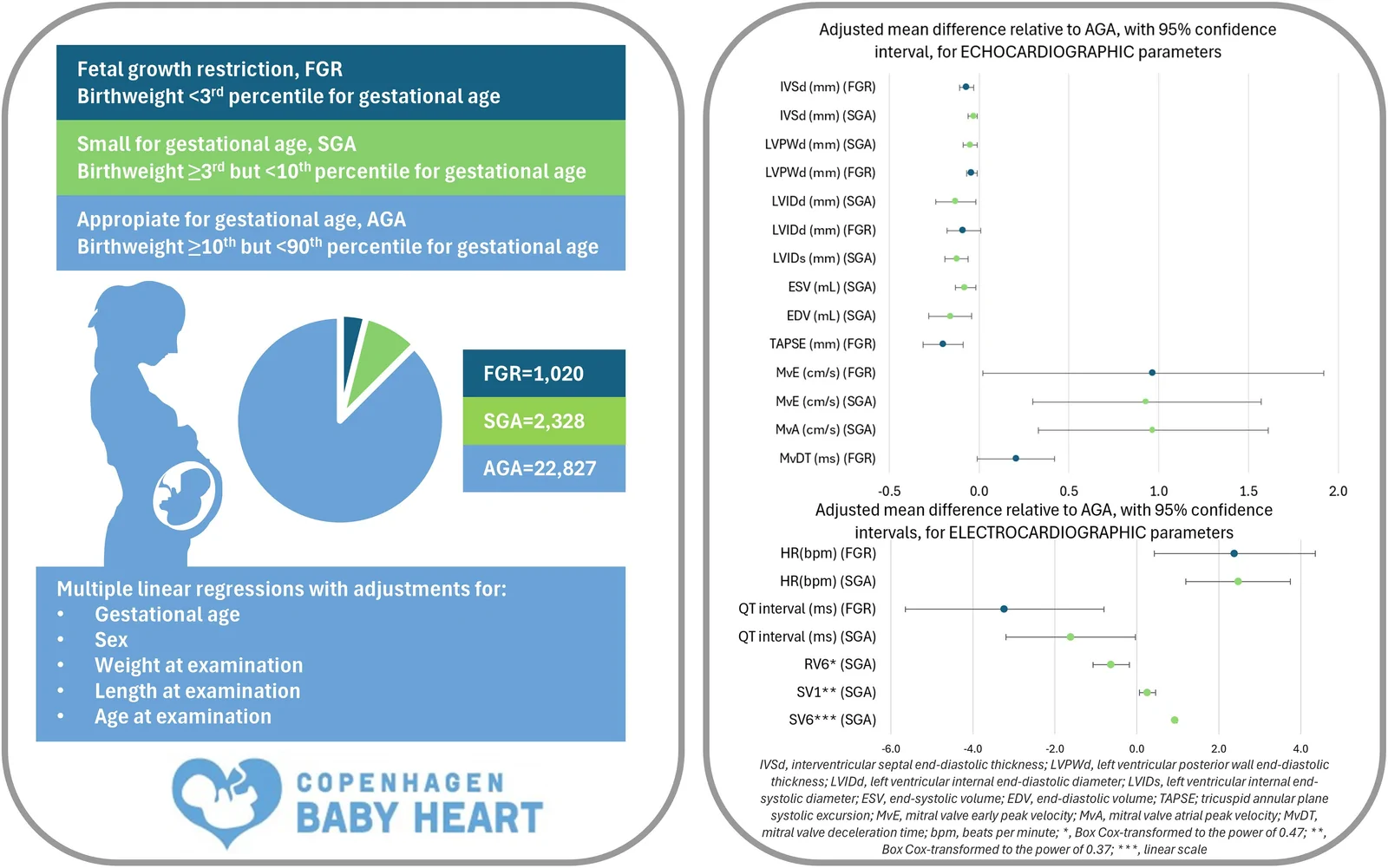

## Introduction

Foetal growth restriction (FGR) and being born small for gestational age (SGA) are both strongly associated with increased neonatal mortality^1,2^ and cardiovascular morbidity later in life.^3,4^ However, whether early stages of cardiovascular dysfunction can be detected early in life is unclear. Smaller echocardiography studies (*n* ≤100 FGR and/or SGA newborns) have suggested that FGR and SGA may be associated with structural changes in the newborn heart, including reduced left ventricular (LV) diameter and reduced thickness of the interventricular septum, and with diastolic alterations such as decreased mitral valve peak velocities.^5–7^ Furthermore, one study of pregnant women with growth-restricted foetuses (*n* = 20) found evidence of alterations in the foetal electrocardiograms, including prolonged QT-intervals.^8^ However, the consequences of FGR and SGA for the newborn cardiac conduction system has never been explored, and newborn cardiac structure and function have not been investigated in a large population-based cohort of newborns.

We used newborn echo- and electrocardiographic measurements from more than 25 000 participants in the Copenhagen Baby Heart Study (CBHS) to investigate the extent to which SGA and FGR are associated with alterations in LV structural and functional parameters and/or the myocardial electrical characteristics in newborns, compared to those born appropriate for gestational age (AGA).

## Methods

### Copenhagen baby heart study

The CBHS is a prospective population-based cohort study that recruited participants from April 2016 until October 2018. All expectant parents planning delivery at one of the three main maternity wards in the Copenhagen area (Herlev Hospital, Hvidovre Hospital, and Rigshospitalet) were invited to enrol their newborns, with the only exclusion criterion being inability to read or speak Danish, English, or Arabic. Parents were invited to participate at the routine ultrasound scan offered to all pregnant women in gestational weeks 18–20. Within 60 days of birth participating newborns were examined using pulse oximetry, transthoracic echocardiography (TTE), and electrocardiography (ECG). Detailed descriptions of the project protocol and the cohort have previously been published.^9,10^

### Study cohort

Those with eligible echocardiographic parameters included all CBHS newborns who had a TTE performed within 60 days of birth, registered weight, and gestational age at birth (*n* = 25 529). Those with eligible ECG parameters included all newborns who had an ECG performed within 30 days of birth (*n* = 17 456). The CBHS’ extensive manual validation of both the echocardiographic and ECG parameters has been described previously.^10,11^

### Exposure

Normal birth weight for gestational age and sex was calculated based on the reference established by Marsal *et al*.^12^ FGR was defined as birth weight for gestational age and sex <3rd percentile, SGA as values between the 3rd and <10th percentiles, AGA as values between the 10th and 90th percentiles, and large for gestational age as values above the 90th percentile. Infants born large for gestational age were excluded from the study (*n* = 2028 in the TTE cohort and *n* = 1452 in the ECG cohort).

### Echocardiographic parameters

TTE was performed on tranquil or sleeping newborns lying in a supine position. The same protocol was used across participating hospitals and included sub-xiphoidal, apical, parasternal, and suprasternal views.^9^ The examinations were performed using 12S-D and 6S-D transducers with Vivid E9 ultrasound equipment (General Electric, Horton, Norway).^9^ Echocardiographic measurements were obtained in accordance with the guidelines for paediatric echocardiography from the American Society of Echocardiograpy.^13^ The following LV parameters were ascertained from two-dimensional echocardiography in the parasternal long-axis view: Interventricular septal end-diastolic thickness (IVSd), LV posterior wall end-diastolic thickness (LVPWd), LV internal diameter in end-diastole (LVIDd), LV internal diameter in end-systole (LVIDs), and fractional shortening. End-diastolic and end-systolic volumes (EDV and ESV), and stroke volume were calculated by the GE Vivid 9 system using Teichholz’s formulae.^14^

Diastolic *trans*-mitral flow velocities were measured using pulsed-wave Doppler in the apical four-chamber view. Trans-mitral flow velocities included mitral valve early peak velocity (MvE), mitral valve atrial peak velocity (MvA), E/A-ratio, and mitral valve deceleration time (MvDT). The apical four-chamber view was also used to assess right ventricular function, measured as the tricuspid annular plane systolic excursion^15^ (TAPSE) as a rough proxy for diastolic function.

### Electrocardiographic parameters

ECGs were obtained and stored using the MAC 5500 DH system (GE ECG System, Milwuakee, USA). Eight-lead ECGs (I, II, III, aVR, AVL, aVF, V1 and V6) were recorded using a paper speed of 25 mm/s, a sensitivity of 10 mm/mV, a sample rate of 500 Hz, and a bandwidth filter of 0.16–150 Hz. ECG traces were stored in the ECG management system MUSE (v8, General Electric Company, Milwuakee, USA). Using GE’s Marquettte 12SL ECG Analysis Program, the ECG parameters were automatically analysed. Validation of the Copenhagen Baby Heart Study’s ECGs has previously found good agreement between manual and automated estimates of ECG parameters.^11^

The following ECG variables were investigated as outcomes in this study: heart rate, PR interval, QRS axis, QRS duration, uncorrected and corrected (Bazett [QTcB]^16^ and Fridericia [QTcF]^17^) QT interval, and maximum amplitude of the R- and S-waves in leads V1 and in the majority of the cohort V6.

### Covariates

We obtained information regarding maternal height, weight, pre-pregnancy body mass index, parity, twin/singleton pregnancy, maternal comorbidities, smoking, and the child’s gestational age at birth, birth weight and birth length from medical records and the internal obstetric database^18^ maintained by the participating hospitals. Maternal comorbidities of interest included pre-existing diabetes [International Classification of Diseases (ICD), version 10, codes O24.0, O24.1, O24.5] and any congenital heart defects (ICD-10 codes Q20.0-Q26.9, Q89.3). We also identified pregnancies complicated by preeclampsia, eclampsia, or the HELLP syndrome, using the ICD-10 codes O14.0-O14.2 and O15.0-O15.9.

### Statistical analyses

We checked the distribution of each set of outcome measurements to ensure that the observed values were approximately normally distributed. The maximum amplitude of the R-wave in V1 and the maximum amplitude of the S-wave in V6 were not normally distributed as measured. Consequently, we used a box-cox transformation on these parameters; optimal normal distributions were obtained by raising the maximum S-wave in V1 to the power of 0.37 and the maximum R-wave in V6 to the power of 0.47.

We removed unrealistic outliers of all electro- and echocardiographic parameters in a two-step process. First, we removed values >10 standard deviations from the mean of each parameter. Afterwards, we removed values >5 standard deviations from the recalculated mean of each parameter.

In our main analyses, we used multiple linear regression to compare echo- and electrocardiographic parameter values in FGR newborns, SGA newborns, and AGA newborns. Models were adjusted for sex, birth weight, birth length, gestational age at birth, and age at examination.

For parameters for which significant changes were identified in the main analyses, we also evaluated the association between SGA or FGR and the risk of extreme echo- and electrocardiographic parameter values by using logistic regression. If the effect estimates from the multiple linear regression model used in the main analyses was negative, we evaluated the association between SGA or FGR and parameter values ≤5th percentile. If the main effect estimate was positive, we evaluated the association with parameter values ≥95th percentile. We adjusted for the same potential confounders as in the main analyses.

To test whether other factors might influence our results, we conducted nine additional sensitivity analyses for both the echo- and electrocardiographic parameters. In four separate analyses, we additionally adjusted for smoking during pregnancy, parity, maternal age, and singleton/twin status. In five additional analyses, we restricted the cohort by excluding: newborns with congenital heart defects, detected during the CBHS’s newborn echocardiographic examination; infants born premature (<37 weeks of gestation); infants born to mothers with pre-existing diabetes; infants born to mothers with congenital heart defects; and infants born to mothers with preeclampsia. Maternal comorbidity was defined using the ICD-10 codes, defined in the Covariates section.

## Ethical approval

The parents of each participant provided written consent to participate in the Copenhagen Baby Heart Study. The Copenhagen Baby Heart Study was approved by the Regional Ethics Committee of the Capital City Region of Denmark (H-16001518) and the Danish Data Protection Agency (I-Suite no: 04546, ID-no. HGH-201653).

## Results

**Figure 1:**
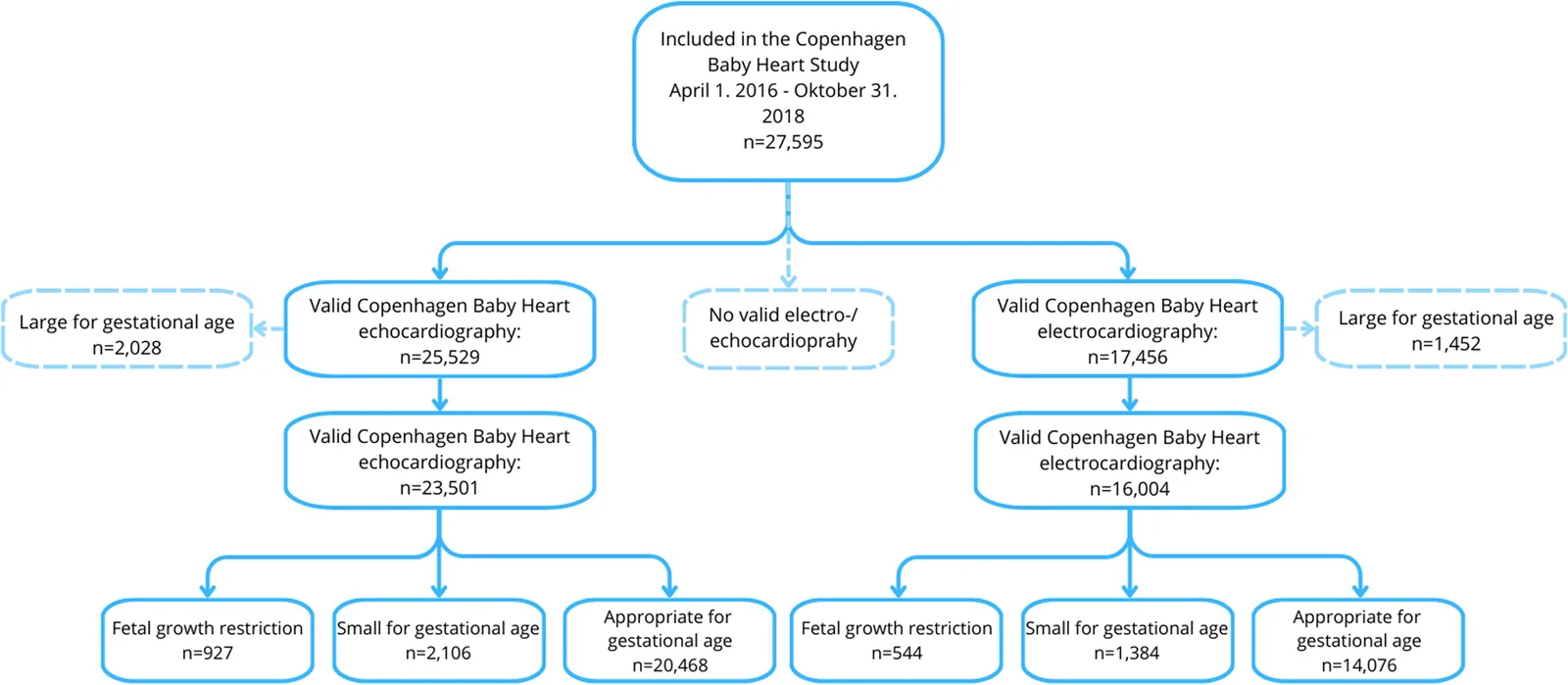
Flow chart illustrating the generation of the study cohorts from the Copenhagen baby heart study, Copenhagen, Denmark, 2016–2018.

For analyses of echocardiographic parameters, our cohort included 927 newborns with FGR, 2106 infants born SGA, and 20 468 infants born AGA (*Figure 1*). Analyses of ECG parameters included 544 newborns with FGR, 1384 infants born SGA, and 14 076 infants born AGA.

*Table 1* shows maternal, pregnancy, delivery, and newborn characteristics of the total cohort used. More FGR newborns than SGA newborns or AGA newborns were born to mothers ≥40 years of age. The prevalence of self-reported maternal smoking in the first trimester was higher for FGR and SGA newborns than for AGA newborns. A larger proportion of newborns with FGR or born SGA than AGA infants were born to nulliparous women and to mothers classified as underweight. Almost twice as many FGR newborns as AGA newborns were delivered by Caesarean section, whereas the prevalence of Caesarean birth among SGA infants born did not differ from that observed in AGA newborns. Newborns with FGR or born SGA were more likely to have been born prematurely than AGA newborns.

**Table 1:** Maternal and newborn characteristics of a cohort of 26 175 newborns from the Copenhagen baby heart study, 2016–2018, by weight for gestational age and sex at birth

	Foetal growth restriction	Small for gestational age	Appropriate for gestational age
	*n* (%)	*n* (%)	*n* (%)
	1020 (3.9)	2328 (8.9)	22 827 (87.2)
**Maternal characteristics**			
Age at delivery (years)			
<25	51 (5.0)	150 (6.4)	1110 (4.9)
25–29	259 (25.4)	643 (27.6)	6627 (29.0)
30–34	400 (39.2)	944 (40.5)	9162 (40.1)
35–39	218 (21.4)	465 (20.0)	4751 (20.8)
≥40	92 (9.0)	126 (5.4)	1177 (5.2)
Pre-pregnancy BMI			
Underweight (<18.5)	65 (6.4)	120 (5.2)	836 (3.7)
Normal weight (18.5–24.9)	657 (64.4)	1583 (68.0)	14 876 (65.2)
Pre-obese (25.0–29.9)	137 (13.4)	317 (13.6)	3838 (16.8)
Obese (≥30.0)	85 (8.3)	135 (5.8)	1673 (7.3)
Missing	76 (7.5)	173 (7.4)	1604 (7.0)
Smoking during pregnancy			
Smoker	59 (5.8)	105 (4.5)	756 (3.3)
Non-smoker	928 (91.0)	2152 (92.4)	21 443 (93.9)
Missing	33 (3.2)	71 (3.0)	628 (2.7)
Parity			
0	761 (74.6)	1654 (71.0)	13 284 (58.2)
1	193 (18.9)	508 (21.8)	7257 (31.8)
2	51 (5.0)	136 (5.8)	1909 (8.4)
≥3	13 (1.3)	29 (1.2)	374 (1.6)
Missing	< 5	< 5	< 5
Known congenital heart defect			
No	1009 (98.9)	2322 (99.7)	22 738 (99.6)
Yes	< 5	< 5	36 (0.2)
Missing	8 (0.8)	< 5	53 (0.2)
Pre-gestational diabetes			
No	1010 (99.0)	2318 (99.6)	22 642 (99.2)
Yes	< 5	8 (0.3)	132 (0.6)
Missing	8 (0.8)	< 5	53 (0.2)
Multiple pregnancy			
No	896 (87.8)	2171 (93.3)	22 435 (98.3)
Yes	124 (12.2)	157 (6.7)	392 (1.7)
Mode of delivery			
Vaginal delivery	719 (70.5)	1935 (83.1)	19 244 (84.3)
Caesarean section	301 (29.5)	393 (16.9)	3583 (15.7)
**Newborn characteristics**			
Sex			
Male	513 (50.3)	1132 (48.6)	11 812 (51.7)
Female			
Gestational age at birth (weeks)			
<35	81 (7.9)	80 (3.4)	283 (1.2)
35	39 (3.8)	24 (1.0)	215 (0.9)
36	72 (7.1)	83 (3.6)	436 (1.9)
37	114 (11.2)	160 (6.9)	1148 (5.0)
38	173 (17.0)	325 (14.0)	2799 (12.3)
39	148 (14.5)	401 (17.2)	4817 (21.1)
40	195 (19.1)	611 (26.2)	6771 (29.7)
≥41	198 (19.4)	644 (27.7)	6358 (27.9)
Birthweight (grams)			
<2500	520 (51.0)	269 (11.6)	351 (1.5)
2500–2999	474 (46.5)	1224 (52.6)	1743 (7.6)
3000–3499	26 (2.5)	835 (35.9)	8581 (37.6)
3500–3999	0 (0.0)	0 (0.0)	9567 (41.9)
≥4000	0 (0.0)	0 (0.0)	2585 (11.3)
Birth length (cm)			
<45	135 (13.2)	63 (2.7)	114 (0.5)
45–49.9	616 (60.4)	1013 (43.5)	2573 (11.3)
50–54.9	246 (24.1)	1215 (52.2)	18 333 (80.3)
>50	< 5	22 (0.9)	1730 (7.6)
Missing	20 (2.0)	15 (0.6)	77 (0.3)
Congenital heart defect			
No	944 (92.6)	2177 (93.5)	21 435 (93.9)
Yes	76 (7.5)	151 (6.5)	1392 (6.1)

### Echocardiographic parameters

*Table 2* shows associations between FGR and SGA, and echocardiographic parameters. After adjusting for weight, length, sex, age at the time of cardiac examination, and gestational age at birth, infants born with FGR or SGA had significantly smaller LV dimensions than infants born AGA, including smaller IVSd, LVPWd, LVIDd, and LVIDs. Newborns born SGA also had smaller end-systolic and end-diastolic LV volumes and smaller stroke volumes. In contrast, there was no evidence of an association between FGR and reductions in LV volumes. Compared with AGA newborns, FGR newborns had a reduced TAPSE [adjusted mean difference (aMD) −0.20 mm, 95% confidence interval (CI) −0.31, −0.09, *P* < 0.001], whereas no reduction in TAPSE was observed for infants born SGA.

**Table 2:** Comparison of left ventricular echocardiographic parameters in infants born with foetal growth restriction or small for gestational age and infants born appropriate for gestational age in a cohort of 25 529 newborns from the Copenhagen baby heart study, 2016–2018

	FGR newborns		SGA newborns	
Echocardiographic parameter	Adjusted mean difference (95% CI)	*P*-value	Adjusted mean difference (95% CI)	*P*-value
Left ventricular structural measurements (mm)				
Interventricular septum end-diastole thickness	−0.07 (−0.11, -0.03)	<0.001	−0.03 (−0.06, -0.01)	0.01
Left ventricular posterior wall end-diastolic thickness	−0.05 (−0.09, −0.01)	0.01	−0.04 (−0.07, −0.01)	<0.01
Left ventricular internal diameter end-diastolic thickness	−0.13 (−0.24, −0.02)	0.03	−0.15 (−0.22, −0.07)	<0.001
Left ventricular internal diameter end-systolic thickness	−0.09 (−0.18, 0.01)	0.06	−0.12 (−0.19, −0.06)	<0.001
Systolic function				
Stroke volume (mL)	−0.02 (−0.15, 0.11)	0.78	−0.08 (−0.17, 0.00)	0.06
Fractional shortening (%)	−0.03 (−0.35, 0.28)	0.84	0.15 (−0.06, 0.36)	0.15
Ejection fraction (%)	−0.03 (−0.46, 0.40)	0.89	0.22 (−0.06, 0.51)	0.13
End-systolic volume (mL)	−0.00 (−0.09, 0.08)	0.97	−0.08 (−0.13, −0.02)	0.01
End-diastolic volume (mL)	−0.03 (−0.21, 0.15)	0.75	−0.16 (−0.28, −0.04)	0.01
TAPSE (mm)	−0.20 (−0.31, −0.09)	<0.001	−0.04 (−0.12, 0.03)	0.27
Diastolic function				
Mitral valve early peak velocity (cm/s)	0.97 (0.02, 1.92)	0.04	0.93 (0.30, 1.57)	<0.01
Mitral valve atrial peak velocity (cm/s)	0.53 (−0.42, 1.48)	0.27	0.97 (0.33, 1.61)	<0.01
Mitral valve deceleration time (ms)	0.21 (−0.01, 0.42)	0.06	−0.04 (−0.18, 0.11)	0.63
E/A ratio	0.01 (−0.02, 0.03)	0.50	−0.00 (−0.02, 0.01)	0.65

CI, confidence interval; TAPSE, Tricuspid Annular Plane Systolic Excursion.

FGR, foetal growth restriction (birthweight <3rd percentile for gestational age and sex)

SGA, small for gestational age (birthweight ≥3rd percentile but <10th percentile for gestational age and sex)

Reference group: infants born appropriate for gestational age (birthweight ≥10th and <90th percentile for gestational age and sex).

All estimates are adjusted for newborn age at the time of cardiac examination, weight, length, and gestational age at birth, and sex.

Newborns with FGR and infants born SGA both exhibited increased early peak flow velocities across the mitral valve (*Table 2*). Infants born SGA also had increased atrial peak flow velocities across the mitral valve, whereas there was a suggested association between FGR and prolonged mitral valve deceleration time (*Table 2*). In contrast, there was no evidence of an association between either FGR or SGA, and E/A ratio (*Table 2*).

### Electrocardiographic parameters

*Table 3* shows associations between FGR, SGA, and newborn ECG parameters. Heart rates were significantly higher for infants born both with FGR or SGA.

**Table 3:** Comparison of electrocardiographic parameters in infants born with foetal growth restriction or small for gestational age and infants born appropriate for gestational age in a cohort of 17 456 newborns from the Copenhagen baby heart study, 2016–2018

	FGR newborns		SGA newborns	
Electrocardiographic parameter ^a^	Adjusted mean difference (95% CI)	*P*-value	Adjusted mean difference (95% CI)	*P*-value
Heart rate (bpm)	2.39 (0.43, 4.35)	0.02	2.48 (1.20, 3.75)	<0.001
QT interval, uncorrected (ms)	−3.23 (−5.65, −0.80)	0.01	−1.61 (−3.19, −0.03)	0.05
QTc Fridericia (ms)	−2.16 (−4.33, 0.02)	0.05	0.02 (−1.40, 1.44)	0.98
QTc Bazett (ms)	−1.20 (−3.55, 1.16)	0.32	1.29 (−0.25, 2.82)	0.10
QRS duration (ms)	−0.41 (−0.98, 0.15)	0.15	−0.36(−0.73, 0.01)	0.06
PR interval (ms)	0.86 (−0.23, 1.94)	0.12	0.10 (−0.6, 0.80)	0.78
QRS axis ^b^	1.00 (0.98, 1.03)	0.70	1.00 (0.98, 1.01)	0.72
Max R-wave amplitude in V1 ^b^	1.00 (0.95, 1.04)	0.87	0.99 (0.96, 1.02)	0.67
Max R-wave amplitude in V6 ^c^	−0.57 (−1.25, 0.12)	0.11	−0.63 (−1.07, −0.18)	0.01
Max S-wave amplitude in V1 ^d^	0.24 (−0.07, 0.55)	0.12	0.26 (0.06, 0.46)	0.01
Max S-wave amplitude in V6 ^b^	0.96 (0.90, 1.04)	0.32	0.93 (0.89, 0.98)	<0.01

bpm, beats per minute; CI, confidence interval; FGR, foetal growth restriction; SGA, small for gestational age.

^a^Due to skewness of some of some outcome variables, transformations were necessary to obtain a normal distribution.

^b^Linear scale. Adjusted mean differences on a linear scale; e.g. 0.93 translates to a reduction of 7%

^c^Boxcox-transformed to the power of 0.47

^d^Boxcox-transformed to the power of 0.37

Reference group: infants born appropriate for gestational age (birthweight ≥10th and <90th percentile).

All estimates are adjusted for newborn age at the time of cardiac examination, weight, length, and gestational age at birth, and sex.

FGR and SGA newborns had shorter uncorrected QT intervals compared with AGA newborns (*Table 3*). However, after correction for heart rate, only FGR was associated with a reduction in QT interval length (QTc Fridericia, aMD −2.16 ms, 95% CI −4.33, 0.02, *P* = 0.05). Our results also suggested that SGA and possibly FGR might also be associated with shorter QRS duration, although neither association was statistically significant (*Table 3*). Otherwise, there was little evidence for an association between either FGR or SGA and PR interval or QRS axis (*Table 3*). Infants born SGA had alterations in the maximum amplitude of the R wave in V6 but not in V1 and in the maximum amplitude of the S wave in both V1 and V6 (*Table 3*). A similar pattern of association was observed for newborns with FGR, although none of the findings were statistically significant (*Table 3*).

### Associations between FGR or SGA and extreme values of echocardiographic and ECG parameters

**Figure 2:**
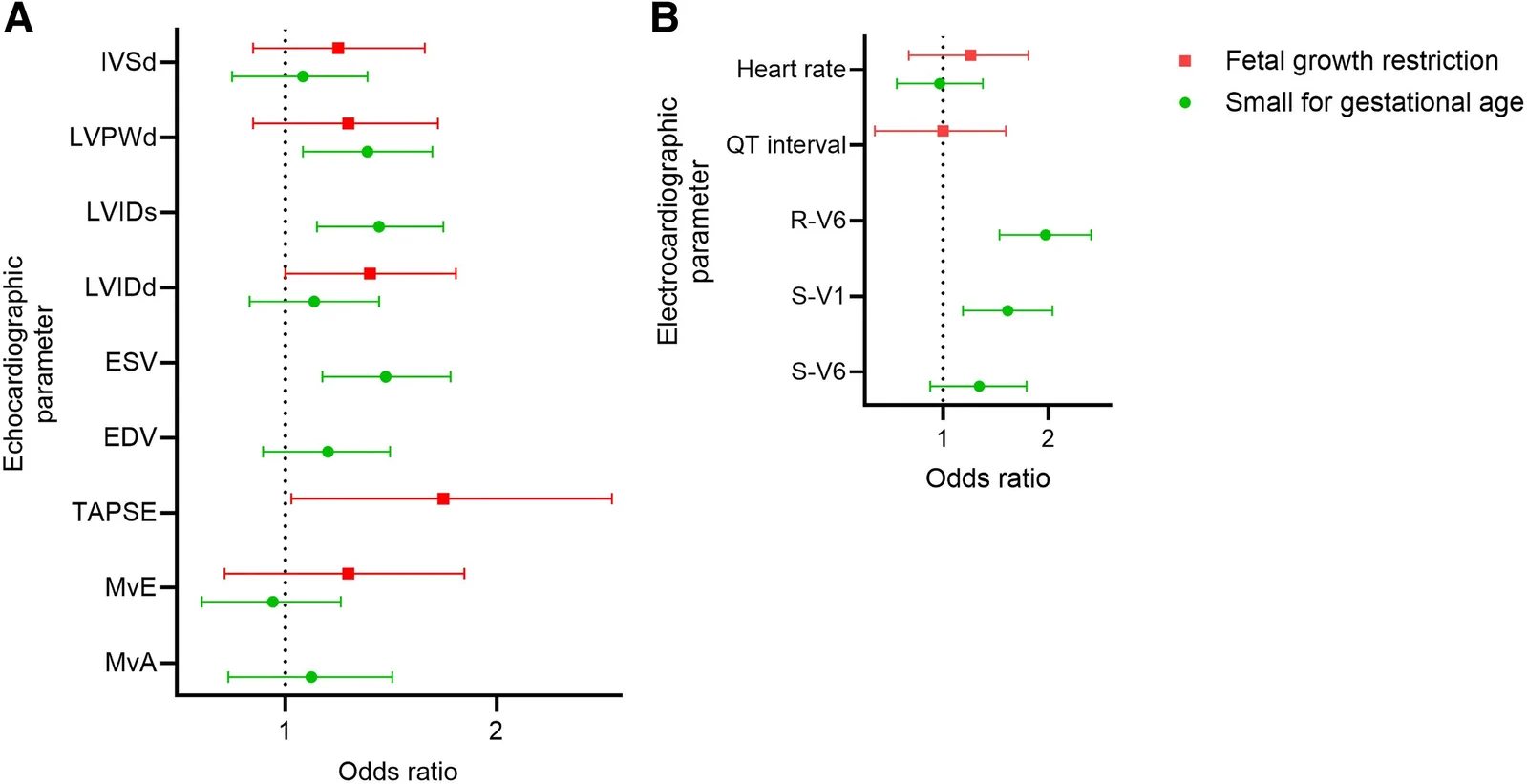
(A and B). Associations between foetal growth restriction, small for gestational age and extreme values of echocardiographic parameters in infancy (2A) (*n* = 25 529), and associations between foetal growth restriction, small for gestational age and extreme values of electrocardiographic parameters in infancy (2B) (*n* = 17 456). Odds ratios with 95% confidence intervals for extreme (≤5th or ≥95th percentile) values of echo- and electrocardiographic parameters comparing newborns with foetal growth restriction (red squares) or born small for gestational age (green circles) with infants born appropriate for gestational age. The direction of the effect estimates from the main linear regression analysis determined whether the ≤5th or ≥95th percentile was evaluated.

*Figure 2A* shows associations between FGR, SGA, and extreme values of LV echocardiographic parameters. Compared with AGA newborns, SGA newborns were more likely to have extremely small (below the 5th percentile) LVPWd, LVIDs and ESV (OR 1.31, 95% CI 1.06, 1.62; OR 1.36, 95% CI 1.11, 1.68; and OR 1.39, 95% CI 1.13, 1.72, respectively). Infants born SGA were not associated with extreme values of IVSd, LVIDd, EDV MvE or MvA (eTable 1). FGR was statistically significantly associated with extremely small values of LVIDd (OR 1.32, 95% CI 1.00, 1.75) and TAPSE (OR 1.68, 95% CI 1.02, 2.92), compared with AGA. Our results also suggested that FGR might be associated with extremely small values of IVSd and LVPWd and extremely large values of MvE, but none of these results was statistically significant (Supplementary  *Table 1*).

*Figure 2B* shows associations between FGR, SGA, and extreme ECG parameter values. There was little evidence of any association between FGR and extreme ECG measurements (Supplementary  *Table 1*). In contrast, SGA newborns were almost twice as likely to have maximum R-wave amplitudes in V6 below the 5th percentile (OR 1.96, 95% CI 1.45, 2.64), and were more than 50% more likely to have maximum S-wave amplitudes in V1 above the 95th percentile (OR 1.53, 95% CI 1.14, 2.05). Neither infants born with FGR or SGA were associated with extreme heart rate values.

### Sensitivity analyses

Results of sensitivity analyses with additional adjustment for potential confounders and various restrictions on the study cohorts are presented in Supplementary  *Tables 2–10*. In most cases, the results did not change meaningfully. However, additional adjustment for multiple pregnancies attenuated the association between FGR and reduction in LVPWd (aMD −0.02 mm, 95% CI −0.06, 0.02, *P* = 0.27) (Supplementary  *Table 5*). Excluding infants born before 37 weeks of gestation from the echocardiography cohort also attenuated the association between FGR and LVPWd (aMD −0.03 mm, 95% CI −0.07, 0.01, *P* = 0.13) (Supplementary  *Table 7*).

## Discussion

### Principal findings

We found that both newborns with FGR and infants born SGA had reduced LV wall thicknesses and left ventricular internal diameters, and both two groups had altered LV diastolic function. Infants born with FGR also had reduced right ventricular function. Furthermore, LV volumes were reduced in infants born SGA. These newborns also had alterations in the precordial leads of the electrocardiogram. Both groups showed signs of reduced QT intervals, particularly newborns with FGR.

### Echocardiographic parameters

Both FGR newborns and SGA newborns exhibited reduced LV wall thicknesses (IVSd and LVPWd) and reduced LV internal diameters. These findings are somewhat in line with findings from previous studies.^6,7,19^ Interestingly, however, Fouzas *et al*.^7^ found that while newborns below the 10th percentile of weight for gestational age examined with TTE two days after birth had reduced LVIDd, internal diameters had normalized 5 days postnatally. In contrast to our findings, Rodriguez-Guerineau *et al*.^6^ only found the septum and posterior wall of the LV to be significantly smaller without adjustment for newborn size, in line with findings from Akazaea *et al*.^5^ In 25 newborns with FGR and 101 infants born SGA, Cinar *et al*.^19^ found no reduction in IVSd thickness. However, the above-mentioned studies were much smaller than ours, and may consequently not have been powered to detect small alterations. In addition, we found that newborns with FGR had significantly increased odds of having extremely small LVIDd, whereas infants born SGA had significantly increased odds of extremely small LVIDs and thin LVPWd.

We found no correlation between fractional shortening or ejection fraction and infants born FGR or with SGA. Similar findings have been reported by previous studies examining systolic function.^5,7^ We found that SGA newborns had reduced end-systolic and end-diastolic volumes and had increased odds of having ESVs in the lowest fifth percentile. Furthermore, they tended to have reduced stroke volumes. In contrast, two previous studies found that newborns with birth weights below the 10th percentile had increased stroke volumes.^6,7^ On the other hand, in line with our findings, Akazawa *et al*. found that newborns with birth weights between the 3rd and 10th percentiles had smaller stroke volumes.^5^ We found little evidence of any association between FGR and altered systolic function.

Both newborns with FGR and infants born SGA had increased values of MvE, with SGA newborns also having increased values of MvA. Persons born with FGR have an increased risk of hypertension later in life.^20–22^ and one study has shown that these newborns already have increased blood pressure in the neonatal period.^23^ Increased values of MvE are associated with increased atrial filling pressure,^24^ suggesting that atrial filling pressure is already increased in FGR and SGA newborns in the neonatal period.

The reduced TAPSE found in infants born with FGR has been observed before.^6^ Rodriguez-Guerineau *et al*. found a slightly reduced TAPSE after adjustment for newborn size. Our results suggest that the previous finding might have been driven by the newborns in the FGR group, as we found no association between SGA and alterations in TAPSE. TAPSE measures the longitudinal contraction of the right ventricle, which is the most profound contraction during the ejection from said ventricle.^25^ In our study, FGR newborns had a 68% increased risk of having extremely small TAPSE values, which could result from diminished right ventricular function, although evidence of changes in other echocardiographic parameters more specific to the function of this ventricle would be required to draw firm conclusions about compromised right ventricular function.

### Electrocardiographic parameters

Compared with infants born AGA, both FGR and SGA newborns had increased heart rates, which has been shown previously.^5,7^ The uncorrected QT interval for both exposure groups was significantly reduced; however, once adjusted for the increased heart rates observed in these groups, the corrected QT intervals were only altered in newborns with FGR.

Infants born SGA had alterations in three of the four precordial measurements. Positive correlations have been found between LV thickness and LV mass index (which account for IVSd and LVPWd) and the amplitudes of the R wave in V6 and the S wave in V1.^26–28^ The observed reduction in amplitude of the R wave in V6 could therefore be a reflection of the reduced LV thicknesses found on TTE in infants born SGA. However, our finding of increased S wave amplitude in V1 cannot be explained by reduced LV thickness. On the other hand, right ventricular hypertrophy is accompanied by reduced S wave amplitudes in V1,^29^ suggesting that observed increases in S wave amplitude might be correlated with reduced right ventricular wall thickness. Persons with hypertension have been shown to have reduced right ventricle internal dimensions^30^; future work should examine whether this is also the case in infants born SGA.

### Sensitivity analyses

Maternal conditions such as preeclampsia and diabetes have been shown to affect both echo- and electrocardiographic parameters in newborns.^31–34^ Congenital heart defects both in the mother and the offspring are also associated with changes in newborns cardiac dimensions and function.^35^ Pregnancy-related factors such as maternal smoking, maternal age, parity, multiple births, and preterm delivery have been linked to changes in the newborn heart as well.^36–38^ Because many of these factors were overrepresented among newborns in our study with FGR or born SGA (compared with infants born AGA), we conducted sensitivity analyses to determine their impact on our results. Most adjustments and exclusions did not affect our results. Additional adjustment for multiple pregnancies and exclusion of infants born before 37 weeks of gestation attenuated the association between FGR and LVPWd. However, excluding preterm newborns resulted in the exclusion of 20% of newborns with FGR from the TTE analyses, which affected the statistical power of our analyses.

### Strengths and potential limitations

Our study had several important strengths. The large size of the study, which was at least an order of magnitude larger than previous studies, allowed us to conclusively establish that FGR and SGA are associated with definite changes in the newborn heart and to uncover some subtle changes in newborn cardiac structure and function. Study personnel performing and analysing the echocardiograms and ECGs were blinded to newborn weight for gestational age status, limiting surveillance bias and differential misclassification of echo- and electrocardiographic parameter values. The population-based nature of the cohort reduced the risk of selection bias.

Potential limitations include an underrepresentation of newborns admitted to the neonatal intensive care unit in the CBHS. As a result, the least severe FGR and SGA phenotypes were probably overrepresented in our study, suggesting that observed differences might have been greater and associations might have been even stronger had the whole spectrum of FGR severity been included. ESV and EDV were estimated from the parasternal long axis view of the echocardiogram, from an apical four-chamber image, which could affect the observed associations, as previous studies have shown that in newborns with FGR, the longitudinal contraction of the LV is most critically affected.

It is well established that FGR can have potentially devastating consequences for the foetus during pregnancy and for the newborn in the immediate postpartum period. However, it is becoming increasingly clear that FGR is an important risk factor for later offspring chronic disease. A recent review by Crispi et. al^39^ documented the long-term cardiovascular morbidity associated with FGR and highlighted the potential importance of early intervention in persons born after FGR in preventing cardiovascular disease in adulthood. Our study suggests that the consequences of FGR may already be visible in the newborn heart, although whether documented long-term cardiovascular disease in persons born with FGR is directly linked with these subclinical alterations in cardiac structure and function present in early infancy is unknown. Further studies of these children are warranted to explore the impact of FGR on cardiac health through childhood and young adulthood, and long-term follow-up of this large cohort of children born FGR will determine whether early management of these children should be considered.

In conclusion, in a large, diverse and well-characterized population of newborns, we found that infants born with foetal growth restriction and infants born small for gestational age had significant alterations in ventricular thickness, diameter, and function, even after adjustment for body size. Furthermore, both groups showed alterations in the electrocardiograms. The increased risks of cardiovascular disease observed in these persons later in life could be a result of the cardiac changes already present in infancy. Follow-up research is needed to understand these changes true effect on cardiovascular outcomes later in life.

## Supplementary Material

oeaf177_Supplementary_Data

## Data Availability

The CBHS welcomes proposals for collaboration and encourages interested parties to contact us at hgh-babyheart@regionh.dk. Per Danish and EU data protection law, CBHS data cannot be uploaded to publicly accessible data repositories. However, data can be made available to specific, well-defined projects upon receipt of approval from the Danish Data Protection Agency, the Scientific Ethics Committees of the Capital City Region of Denmark, and the CBHS’ steering committee.
