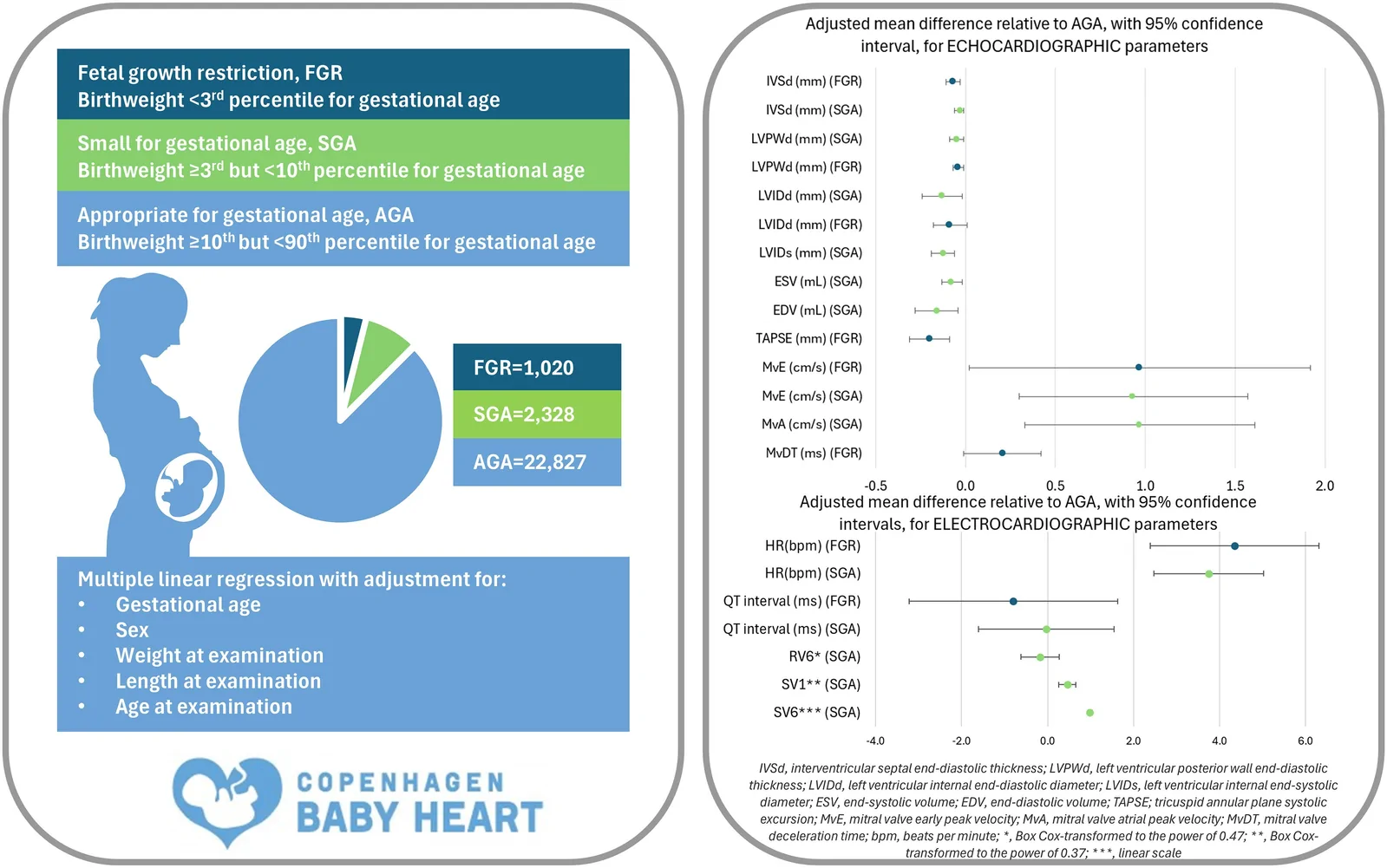# Correction to: Impact of foetal growth restriction and being born small for gestational age on newborn echo- and electrocardiographic measurements—a Copenhagen baby heart study

**DOI:** 10.1093/ehjopen/oeag129

**Published:** 2026-08-21

This is a correction to: Emil H Nørskov, Signe L Skjellerup, Johan E Navne, Raheel A Raja, Maria M Pærregaard, Anne-Sophie Sillesen, Anna A Raja, Alex H Christensen, Kasper K Iversen, Henning Bundgaard, Heather A Boyd, Dorthe L Jeppesen, R Ottilia B Vøgg, Impact of foetal growth restriction and being born small for gestational age on newborn echo- and electrocardiographic measurements—a Copenhagen baby heart study, *European Heart Journal Open*, Volume 6, Issue 1, January 2026, oeaf177, https://doi.org/10.1093/ehjopen/oeaf177

In the originally published version of this manuscript's graphical abstract, the values under the ‘Adjusted mean difference relative to AGA, with 95% confidence intervals, for ELECTROCARDIOGRAPHIC parameters’ heading were erroneously centred around the upper 95% confidence interval rather than the adjusted mean.

This error has been corrected so that the graphical abstract now reads:

**Figure:**
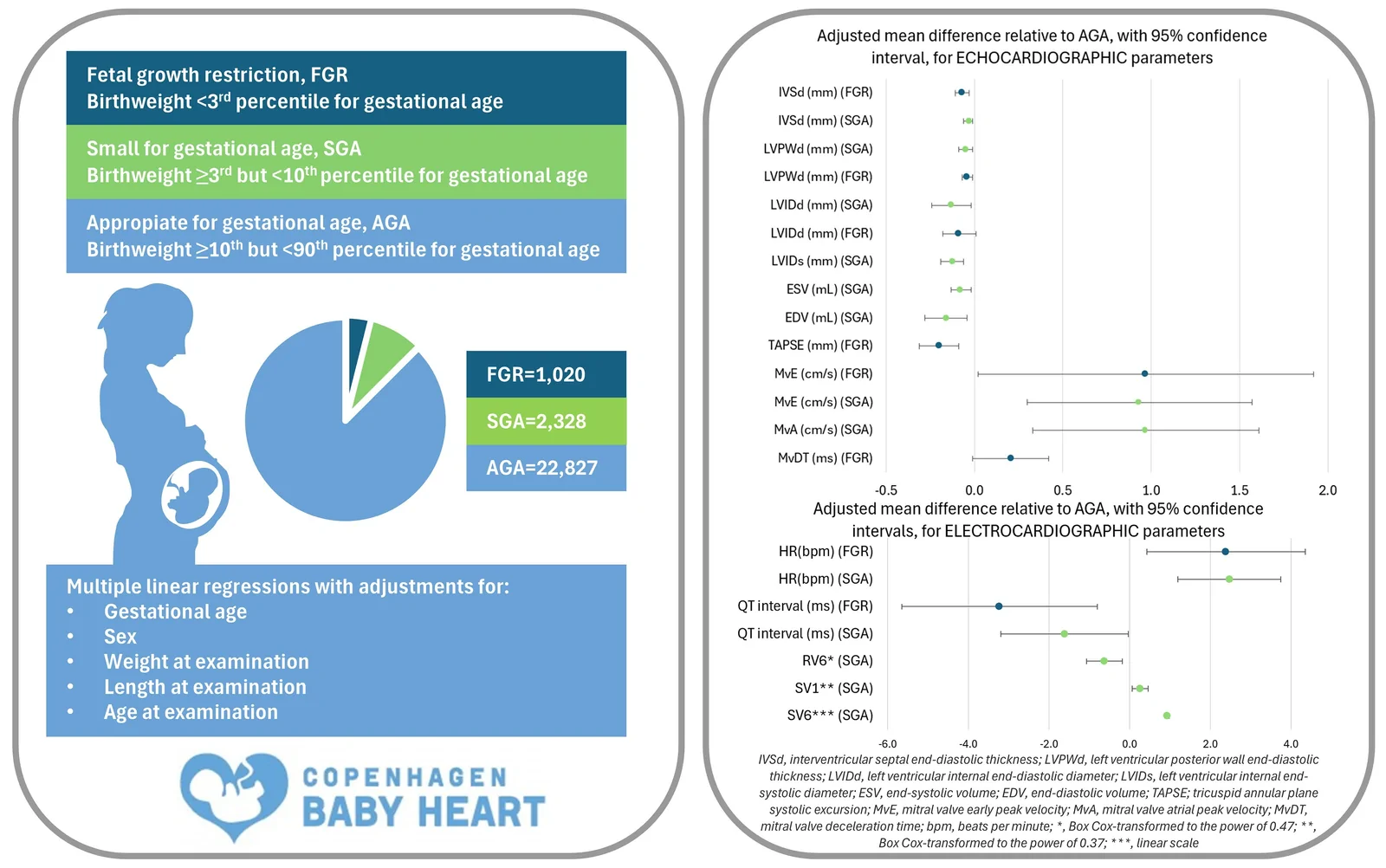


Instead of:

**Figure:**