# Supplementary material for: Impact of foetal growth restriction and being born small for gestational age on newborn echo- and electrocardiographic measurements—a Copenhagen baby heart study
Source: Eur Heart J Open. 2026 Jan 7;6(1):oeaf177. doi: 10.1093/ehjopen/oeaf177 (PMC12825605; doi:10.1093/ehjopen/oeaf177)
Supplement: oeaf177_Supplementary_Data [file oeaf177_supplementary_data.docx]

eTable 1. Odds ratios of extreme values used in Figure 2 and 3

| Variable | Estimate | Lower leg | Upper leg |
| --- | --- | --- | --- |
| **Echocardiographic parameters** | | | |
| Fetal Growth Restriction | | | |
| Interventricular septum end-diastole thickness | 1.19 | 0.90 | 1.58 |
| Left ventricular posterior wall end-diastolic thickness | 1.23 | 0.90 | 1.65 |
| Left ventricular internal diameter in diastole | 1.32 | 1.00 | 1.75 |
| [Tricuspid Annular Plane Systolic Excursion](https://soeg.kb.dk/discovery/fulldisplay?docid=cdi_proquest_miscellaneous_1675876912&context=PC&vid=45KBDK_KGL:KGL&lang=da&search_scope=MyInst_and_CI&adaptor=Primo%20Central&tab=Everything&query=any%2Ccontains%2CTricuspid%20annular%20plane%20systolic%20excursion%20is%20reduced%20in%20infants%20with%20pulmonary%20hypertension%3A%20value%20of%20tricuspid%20annular%20plane%20systolic%20excursion%20(TAPSE)%20to%20determine%20right%20ventricular%20function%20in%20various%20conditions%20of%20pediatric%20pulmonary%20hypertension) | 1.68 | 1.02 | 2.92 |
| Mitral valve early peak velocity | 1.23 | 0.82 | 1.80 |
| Small for Gestational Age | | | |
| Interventricular septum end-diastole thickness | 1.06 | 0.84 | 1.31 |
| Left ventricular posterior wall end-diastolic thickness | 1.31 | 1.06 | 1.62 |
| Left ventricular internal diameter in diastole | 1.10 | 0.89 | 1.36 |
| Left ventricular internal diameter in systole | 1.36 | 1.11 | 1.68 |
| End-systolic volume | 1.39 | 1.13 | 1.72 |
| End-diastolic volume | 1.15 | 0.93 | 1.41 |
| Mitral valve early peak velocity | 0.96 | 0.76 | 1.20 |
| Mitral valve atrial peak velocity | 1.09 | 0.83 | 1.42 |
| **Electrocardiographic parameters** | | | |
| Fetal Growth Restriction | | | |
| Heart Rate | 1.20 | 0.80 | 1.75 |
| QT interval | 1.00 | 0.64 | 1.51 |
| Small for Gestational Age | | | |
| Heart Rate | 0.98 | 0.74 | 1.30 |
| R-wave amplitude in V6 | 1.96 | 1.45 | 2.64 |
| S-wave amplitude in V1 | 1.53 | 1.14 | 2.05 |
| S-wave amplitude in V6 | 1.27 | 0.92 | 1.73 |

Odds ratios calculated using logistic regression of significant values from Tables 1 and 2 used for the forrest plots in Figure 2 and 3.

eTable 2. Comparison of echocardiographic left ventricular measures and electrocardiographic measurements in infants born with fetal growth restriction or small for gestational age compared with infants born appropriate for gestational age.

Sensitivity analyses with further adjustment for smoking in addition to the adjustments in the main analyses.

|  | Infants born with Fetal Growth Restriction (n=927) | | | Infants born Small for Gestational Age (n=2,106) | | |
| --- | --- | --- | --- | --- | --- | --- |
| Echocardiographic parameter | Mean adjusted difference (95% CI) | | p-value | Mean adjusted difference (95% CI) | | p-value |
| E/A ratio | 0.01 | (-0.02, 0.03) | 0.57 | 0.00 | (-0.02, 0.01) | 0.62 |
| Stroke Volume (mL) | -0.02 | (-0.16, 0.11) | 0.71 | -0.09 | (-0.18, 0.00) | 0.04 |
| Fractional Shortening (%) | -0.08 | (-0.40, 0.23) | 0.61 | 0.14 | (-0.07, 0.36) | 0.18 |
| Ejection Fraction (%) | -0.10 | (-0.54, 0.33) | 0.64 | 0.21 | (-0.08, 0.50) | 0.16 |
| Mitral valve early peak velocity (cm/s) | 0.98 | (0.02, 1.94) | 0.05 | 0.85 | (0.20, 1.50) | 0.01 |
| Mitral valve atrial peak velocity (cm/s) | 0.62 | (-0.34, 1.58) | 0.21 | 0.92 | (0.27, 1.56) | 0.01 |
| Mitral valve deceleration time (ms) | 0.20 | (-0.02, 0.42) | 0.07 | -0.02 | (-0.17, 0.12) | 0.77 |
| Left ventricular posterior wall end-diastolic thickness (mm) | -0.05 | (-0.09, -0.01) | 0.01 | -0.04 | (-0.06, -0.01) | <0.01 |
| Left ventricular internal diameter end-diastolic thickness (mm) | -0.12 | (-0.23, -0.01) | 0.04 | -0.15 | (-0.23, -0.08) | <0.001 |
| Left ventricular internal diameter end-systolic thickness(mm) | -0.07 | (-0.17, 0.02) | 0.13 | -0.13 | (-0.19, -0.06) | <0.001 |
| Interventricular septum end-diastole thickness (mm) | -0.08 | (-0.11, -0.04) | <0.001 | -0.03 | (-0.06, -0.01) | 0.01 |
| End-systolic volume (mL) | 0.01 | (-0.07, 0.10) | 0.76 | -0.08 | (-0.13, -0.02) | 0.01 |
| End-diastolic volume (mL) | -0.02 | (-0.21, 0.17) | 0.84 | -0.17 | (-0.29, -0.04) | 0.01 |
| TAPSE (mm) | -0.21 | (-0.32, -0.10) | <0.001 | -0.05 | (-0.13, 0.03) | 0.19 |
|  |  | | |  | | |
| Electrocardiographic parameters | Infants born with Fetal Growth Restriction (n=544) | | | Infants born Small for Gestational Age (n=1,384) | | |
| Heart rate (bpm) | 2.33 | (0.35, 4.32) | 0.02 | 2.40 | (1.11, 3.69) | <0.001 |
| QT interval, uncorrected (ms) | -3.22 | (-5.68, -0.76) | 0.01 | -1.49 | (-3.09, 0.12) | 0.07 |
| QTc Fridericia (ms) | -2.18 | (-4.39, 0.03) | 0.05 | 0.15 | (-1.29, 1.59) | 0.84 |
| QTc Bazett (ms) | -1.25 | (-3.64, 1.14) | 0.31 | 1.41 | (-0.14, 2.97) | 0.08 |
| QRS duration (ms) | -0.38 | (-0.95, 0.19) | 0.19 | -0.34 | (-0.72, 0.03) | 0.07 |
| PR interval (ms) | 0.84 | (-0.26, 1.94) | 0.13 | 0.02 | (-0.69, 0.73) | 0.95 |
| QRS axis ^b^ | 1.00 | (0.98 ,1.03) | 0.71 | 1.00 | (0.98, 1.01) | 0.72 |
| Max R-wave amplitude in V1 ^b^ | 1.00 | (0.95, 1.04) | 0.85 | 0.99 | (0.96, 1.02) | 0.63 |
| Max R-wave amplitude in V6 ^c^ | -0.63 | (-1.33, 0.06) | 0.07 | -0.61 | (-1.06, -0.16) | 0.01 |
| Max S-wave amplitude in V1 ^d^ | 0.30 | (-0.02, 0.61) | 0.06 | 0.25 | (0.04, 0.45) | 0.02 |
| Max S-wave amplitude in V6 ^b^ | 0.96 | (0.90, 1.03) | 0.28 | 0.93 | (0.89, 0.98) | <0.01 |
|  |  |  |  |  |  |  |

Bpm; beats per minute; CI; confidence interval, ms; milliseconds, TAPSE; [Tricuspid Annular Plane Systolic Excursion](https://soeg.kb.dk/discovery/fulldisplay?docid=cdi_proquest_miscellaneous_1675876912&context=PC&vid=45KBDK_KGL:KGL&lang=da&search_scope=MyInst_and_CI&adaptor=Primo%20Central&tab=Everything&query=any%2Ccontains%2CTricuspid%20annular%20plane%20systolic%20excursion%20is%20reduced%20in%20infants%20with%20pulmonary%20hypertension%3A%20value%20of%20tricuspid%20annular%20plane%20systolic%20excursion%20(TAPSE)%20to%20determine%20right%20ventricular%20function%20in%20various%20conditions%20of%20pediatric%20pulmonary%20hypertension).

^a^ Due to skewness of some of some outcome variables, transformations were necessary to obtain a normal distribution.

^b^ Linear scale. Adjusted mean differences on a linear scale; eg. 0.93 translates to a reduction of 7%

^c^ Boxcox-transformed to the power of 0.47

^d^ Boxcox-transformed to the power of 0.37

Reference group: infants born appropriate for gestational age (echocardiography: n=20,468, electrocardiography: n=14,076) [birthweight ≥10^th^ and <90^th^ percentile]).

All estimates are adjusted for newborn age at the time of cardiac examination, weight, length, and gestational age at birth, and sex.

eTable 3. Comparison of echocardiographic left ventricular measures and electrocardiographic measurements in infants born with fetal growth restriction or small for gestational age compared with infants born appropriate for gestational age.

Sensitivity analyses with further adjustment for parity

|  | Infants born with Fetal Growth Restriction (n=927) | | | Infants born Small for Gestational Age (n=2,106) | | |
| --- | --- | --- | --- | --- | --- | --- |
| Variable | Mean adjusted difference (95% CI) | | p-value | Mean adjusted difference (95% CI) | | p-value |
| E/A ratio | 0.01 | (-0.02, 0.03) | 0.52 | 0.00 | (-0.02, 0.01) | 0.64 |
| Stroke Volume (mL) | -0.02 | (-0.15, 0.11) | 0.77 | -0.08 | (-0.17, 0.00) | 0.06 |
| Fractional Shortening (%) | -0.05 | (-0.37, 0.26) | 0.74 | 0.15 | (-0.06, 0.36) | 0.16 |
| Ejection Fraction (%) | -0.06 | (-0.49, 0.37) | 0.79 | 0.22 | (-0.07, 0.50) | 0.14 |
| Mitral valve early peak velocity (cm/s) | 1.08 | (0.13, 2.03) | 0.03 | 0.97 | (0.33, 1.61) | <0.01 |
| Mitral valve atrial peak velocity (cm/s) | 0.66 | (-0.29, 1.61) | 0.17 | 1.01 | (0.37, 1.65) | <0.01 |
| Mitral valve deceleration time (ms) | 0.20 | (-0.02, 0.41) | 0.07 | -0.04 | (-0.18, 0.11) | 0.59 |
| Left ventricular posterior wall end-diastolic thickness (mm) | -0.05 | (-0.09, -0.01) | 0.01 | -0.04 | (-0.07, -0.01) | <0.01 |
| Left ventricular internal diameter end-diastolic thickness (mm) | -0.12 | (-0.24, -0.01) | 0.03 | -0.14 | (-0.22, -0.07) | <0.001 |
| Left ventricular internal diameter end-systolic thickness(mm) | -0.08 | (-0.18, 0.01) | 0.09 | -0.12 | (-0.19, -0.06) | <0.001 |
| Interventricular septum end-diastole thickness (mm) | -0.07 | (-0.11, -0.03) | <0.001 | -0.03 | (-0.06, -0.01) | 0.01 |
| End-systolic volume (mL) | 0.00 | (-0.08, 0.09) | 0.93 | -0.07 | (-0.13, -0.02) | 0.01 |
| End-diastolic volume (mL) | -0.03 | (-0.21, 0.16) | 0.79 | -0.16 | (-0.28, -0.03) | 0.01 |
| TAPSE (mm) | -0.20 | (-0.31, -0.08) | <0.001 | -0.04 | (-0.12, 0.03) | 0.25 |
|  |  |  |  |  |  |  |
| Electrocardiographic parameters | Infants born with Fetal Growth Restriction (n=544) | | | Infants born Small for Gestational Age (n=1,384) | | |
| Heart rate (bpm) | 2.42 | (0.46, 4.38) | 0.02 | 2.51 | (1.24, 3.78) | <0.001 |
| QT interval, uncorrected (ms) | -3.32 | (-5.74, -0.90) | 0.01 | -1.70 | (-3.28, -0.12) | 0.03 |
| QTc Fridericia (ms) | -2.23 | (-4.41, -0.06) | 0.04 | -0.05 | (-1.47, 1.37) | 0.95 |
| QTc Bazett (ms) | -1.25 | (-3.61, 1.10) | 0.30 | 1.23 | (-0.30, 2.77) | 0.12 |
| QRS duration (ms) | -0.43 | (-0.99, 0.14) | 0.14 | -0.37 | (-0.74, 0.00) | 0.05 |
| PR interval (ms) | 0.85 | (-0.24, 1.94) | 0.12 | 0.09 | (-0.61, 0.80) | 0.79 |
| QRS axis ^b^ | 1.00 | (0.98, 1.03) | 0.70 | 1.00 | (0.98, 1.01) | 0.70 |
| Max R-wave amplitude in V1 ^b^ | 1.00 | (0.95, 1.04) | 0.87 | -0.99 | (0.96, 1.02) | 0.67 |
| Max R-wave amplitude in V6 ^c^ | -0.55 | (-1.24, 0.13) | 0.12 | -0.61 | (-1.05, -0.16) | 0.01 |
| Max S-wave amplitude in V1 ^d^ | 0.24 | (-0.07, 0.55) | 0.12 | 0.26 | (0.06, 0.46) | 0.01 |
| Max S-wave amplitude in V6 ^b^ | 0.96 | (0.90, 1.04) | 0.33 | 0.93 | (0.89, 0.98) | <0.01 |

Bpm; beats per minute; CI; confidence interval, ms; milliseconds, TAPSE; [Tricuspid Annular Plane Systolic Excursion](https://soeg.kb.dk/discovery/fulldisplay?docid=cdi_proquest_miscellaneous_1675876912&context=PC&vid=45KBDK_KGL:KGL&lang=da&search_scope=MyInst_and_CI&adaptor=Primo%20Central&tab=Everything&query=any%2Ccontains%2CTricuspid%20annular%20plane%20systolic%20excursion%20is%20reduced%20in%20infants%20with%20pulmonary%20hypertension%3A%20value%20of%20tricuspid%20annular%20plane%20systolic%20excursion%20(TAPSE)%20to%20determine%20right%20ventricular%20function%20in%20various%20conditions%20of%20pediatric%20pulmonary%20hypertension).

^a^ Due to skewness of some of some outcome variables, transformations were necessary to obtain a normal distribution.

^b^ Linear scale. Adjusted mean differences on a linear scale; eg. 0.93 translates to a reduction of 7%

^c^ Boxcox-transformed to the power of 0.47

^d^ Boxcox-transformed to the power of 0.37

Reference group: infants born appropriate for gestational age (echocardiography: n=20,468, electrocardiography: n=14,076) [birthweight ≥10^th^ and <90^th^ percentile]).

All estimates are adjusted for newborn age at the time of cardiac examination, weight, length, and gestational age at birth, and sex.

eTable 4. Comparison of echocardiographic left ventricular measures and electrocardiographic measurements in infants born with fetal growth restriction or small for gestational age compared with infants born appropriate for gestational age.

Sensitivity analyses with further adjustment for maternal age

|  | Infants born with Fetal Growth Restriction (n=927) | | | Infants born Small for Gestational Age (n=2,106) | | |
| --- | --- | --- | --- | --- | --- | --- |
| Variable | Mean adjusted difference (95% CI) | | p-value | Mean adjusted difference (95% CI) | | p-value |
| E/A ratio | 0.01 | (-0.02, 0.03) | 0.50 | 0.00 | (-0.02, 0.01) | 0.65 |
| Stroke Volume (mL) | -0.02 | (-0.15, 0.11) | 0.78 | -0.08 | (-0.17, 0.00) | 0.06 |
| Fractional Shortening (%) | -0.03 | (-0.35, 0.28) | 0.84 | 0.15 | (-0.05, 0.36) | 0.15 |
| Ejection Fraction (%) | -0.03 | (-0.46, 0.40) | 0.88 | 0.22 | (-0.06, 0.51) | 0.13 |
| Mitral valve early peak velocity (cm/s) | 0.97 | (0.02, 1.91) | 0.05 | 0.94 | (0.30, 1.58) | <0.01 |
| Mitral valve atrial peak velocity (cm/s) | 0.53 | (-0.42, 1.48) | 0.28 | 0.98 | (0.34, 1.62) | <0.01 |
| Mitral valve deceleration time (ms) | 0.21 | (-0.01, 0.42) | 0.06 | -0.04 | (-0.18, 0.11) | 0.63 |
| Left ventricular posterior wall end-diastolic thickness (mm) | -0.05 | (-0.09, -0.01) | 0.01 | -0.04 | (-0.07, -0.01) | <0.01 |
| Left ventricular internal diameter end-diastolic thickness (mm) | -0.13 | (-0.24, -0.02) | 0.03 | -0.15 | (-0.22, -0.07) | <0.001 |
| Left ventricular internal diameter end-systolic thickness(mm) | -0.09 | (-0.18, 0.01) | 0.07 | -0.12 | (-0.19, -0.06) | <0.001 |
| Interventricular septum end-diastole thickness (mm) | -0.07 | (-0.11, -0.03) | <0.001 | -0.03 | (-0.06, -0.01) | 0.01 |
| End-systolic volume (mL) | 0.00 | (-0.09, 0.08) | 0.98 | -0.08 | (-0.13, -0.02) | 0.01 |
| End-diastolic volume (mL) | -0.03 | (-0.21, 0.15) | 0.76 | -0.16 | (-0.28, -0.04) | 0.01 |
| TAPSE (mm) | -0.20 | (-0.31, -0.09) | <0.001 | -0.04 | (-0.12, 0.03) | 0.25 |
|  |  |  |  |  |  |  |
| Electrocardiographic parameters | Infants born with Fetal Growth Restriction (n=544) | | | Infants born Small for Gestational Age (n=1,384) | | |
| Heart rate (bpm) | 2.40 | (0.44, 4.36) | 0.02 | 2.47 | (1.19, 3.74) | <0.001 |
| QT interval, uncorrected (ms) | -3.24 | (-5.67, -0.82) | 0.01 | -1.58 | (-3.16, 0.00) | 0.05 |
| QTc Fridericia (ms) | -2.17 | (-4.35, 0.00) | 0.05 | 0.04 | (-1.38, 1.46) | 0.95 |
| QTc Bazett (ms) | -1.21 | (-3.56, 1.15) | 0.32 | 1.30 | (-0.24, 2.84) | 0.10 |
| QRS duration (ms) | -0.42 | (-0.98, 0.15) | 0.15 | -0.36 | (-0.72, 0.01) | 0.06 |
| PR interval (ms) | 0.86 | (-0.23, 1.95) | 0.12 | 0.10 | (-0.61, 0.80) | 0.78 |
| QRS axis ^b^ | 1.00 | (0.98, 1.03) | 0.66 | 1.00 | (0.98, 1.01) | 0.65 |
| Max R-wave amplitude in V1 ^b^ | 1.00 | (0.95, 1.05) | 0.90 | 0.99 | (0.96, 1.02) | 0.63 |
| Max R-wave amplitude in V6 ^c^ | -0.57 | (-1.25, 0.12) | 0.11 | -0.62 | (-1.07, -0.18) | 0.01 |
| Max S-wave amplitude in V1 ^d^ | 0.24 | (-0.07, 0.55) | 0.13 | 0.26 | (0.06, 0.47) | 0.01 |
| Max S-wave amplitude in V6 ^b^ | 0.96 | (0.90, 1.04) | 0.32 | 0.93 | (0.89, 0.98) | <0.01 |

Bpm; beats per minute; CI; confidence interval, ms; milliseconds, TAPSE; [Tricuspid Annular Plane Systolic Excursion](https://soeg.kb.dk/discovery/fulldisplay?docid=cdi_proquest_miscellaneous_1675876912&context=PC&vid=45KBDK_KGL:KGL&lang=da&search_scope=MyInst_and_CI&adaptor=Primo%20Central&tab=Everything&query=any%2Ccontains%2CTricuspid%20annular%20plane%20systolic%20excursion%20is%20reduced%20in%20infants%20with%20pulmonary%20hypertension%3A%20value%20of%20tricuspid%20annular%20plane%20systolic%20excursion%20(TAPSE)%20to%20determine%20right%20ventricular%20function%20in%20various%20conditions%20of%20pediatric%20pulmonary%20hypertension).

^a^ Due to skewness of some of some outcome variables, transformations were necessary to obtain a normal distribution.

^b^ Linear scale. Adjusted mean differences on a linear scale; eg. 0.93 translates to a reduction of 7%

^c^ Boxcox-transformed to the power of 0.47

^d^ Boxcox-transformed to the power of 0.37

Reference group: infants born appropriate for gestational age (echocardiography: n=20,468, electrocardiography: n=14,076) [birthweight ≥10^th^ and <90^th^ percentile]).

All estimates are adjusted for newborn age at the time of cardiac examination, weight, length, and gestational age at birth, and sex.

eTable 5. Comparison of echocardiographic left ventricular measures and electrocardiographic measurements in infants born with fetal growth restriction or small for gestational age compared with infants born appropriate for gestational age.

Sensitivity analyses with further adjustment for twin births.

|  | Infants born with Fetal Growth Restriction (n=927) | | | Infants born Small for Gestational Age (n=2,106) | | |
| --- | --- | --- | --- | --- | --- | --- |
| Variable | Mean adjusted difference (95% CI) | | p-value | Mean adjusted difference (95% CI) | | p-value |
| E/A ratio | 0.01 | (-0.02, 0.03) | 0.60 | -0.01 | (-0.03, 0.01) | 0.26 |
| Stroke Volume (mL) | -0.04 | (-0.18, 0.11) | 0.62 | -0.09 | (-0.19, 0.00) | 0.06 |
| Fractional Shortening (%) | -0.12 | (-0.46, 0.22) | 0.50 | 0.13 | (-0.10, 0.36) | 0.26 |
| Ejection Fraction (%) | -0.15 | (-0.61, 0.32) | 0.53 | 0.18 | (-0.13, 0.49) | 0.25 |
| Mitral valve early peak velocity (cm/s) | 0.82 | (-0.24, 1.88) | 0.13 | 0.96 | (0.24, 1.67) | 0.01 |
| Mitral valve atrial peak velocity (cm/s) | 0.47 | (-0.58, 1.52) | 0.38 | 1.30 | (0.59, 2.01) | <0.01 |
| Mitral valve deceleration time (ms) | 0.04 | (-0.21, 0.28) | 0.76 | -0.06 | (-0.22, 0.11) | 0.51 |
| Left ventricular posterior wall end-diastolic thickness (mm) | -0.02 | (-0.06, 0.02) | 0.27 | -0.03 | (-0.06, 0.00) | 0.03 |
| Left ventricular internal diameter end-diastolic thickness (mm) | -0.15 | (-0.27, -0.02) | 0.02 | -0.14 | (-0.22, -0.06) | <0.01 |
| Left ventricular internal diameter end-systolic thickness(mm) | -0.08 | (-0.19, 0.02) | 0.11 | -0.11 | (-0.18, -0.04) | <0.01 |
| Interventricular septum end-diastole thickness (mm) | -0.05 | (-0.09, -0.01) | 0.02 | -0.03 | (-0.06, -0.01) | 0.01 |
| End-systolic volume (mL) | 0.00 | (-0.09, 0.09) | 1.00 | -0.07 | (-0.14, -0.01) | 0.02 |
| End-diastolic volume (mL) | -0.07 | (-0.28, 0.13) | 0.48 | -0.16 | (-0.30, -0.03) | 0.02 |
| TAPSE (mm) | -0.16 | (-0.28, -0.04) | 0.01 | -0.04 | (-0.13, 0.04) | 0.30 |
|  |  |  |  |  |  |  |
| Electrocardiographic parameters | Infants born with Fetal Growth Restriction (n=544) | | | Infants born Small for Gestational Age (n=1,384) | | |
| Heart rate (bpm) | 2.54 | (0.47, 4.6) | 0.02 | 2.48 | (1.15, 3.81) | <0.001 |
| QT interval, uncorrected (ms) | -3.24 | (-5.79, -0.69) | 0.01 | -1.51 | (-3.16, 0.15) | 0.07 |
| QTc Fridericia (ms) | -2.01 | (-4.29, 0.28) | 0.08 | 0.15 | (-1.33, 1.63) | 0.84 |
| QTc Bazett (ms) | -0.93 | (-3.40, 1.53) | 0.46 | 1.43 | (-0.17, 3.03) | 0.08 |
| QRS duration (ms) | -0.42 | (-1.00, 0.16) | 0.16 | -0.39 | (-0.76, -0.01) | 0.04 |
| PR interval (ms) | 1.11 | (-0.01, 2.22) | 0.05 | 0.27 | (-0.45, 0.99) | 0.47 |
| QRS axis ^b^ | 1.01 | (0.99, 1.03) | 0.36 | 1.00 | (0.99, 1.01) | 0.91 |
| Max R-wave amplitude in V1 ^b^ | 1.01 | (0.95, 1.06) | 0.83 | 1.00 | (0.97, 1.03) | 0.92 |
| Max R-wave amplitude in V6 ^c^ | -0.59 | (-1.28, 0.10) | 0.09 | -0.64 | (-1.09, -0.19) | 0.01 |
| Max S-wave amplitude in V1 ^d^ | 0.28 | (-0.05, 0.60) | 0.09 | 0.25 | (0.04, 0.46) | 0.02 |
| Max S-wave amplitude in V6 ^b^ | -0.96 | (0.90, 1.03) | 0.31 | 0.93 | (0.89, 0.97) | <0.01 |

Bpm; beats per minute; CI; confidence interval, ms; milliseconds, TAPSE; [Tricuspid Annular Plane Systolic Excursion](https://soeg.kb.dk/discovery/fulldisplay?docid=cdi_proquest_miscellaneous_1675876912&context=PC&vid=45KBDK_KGL:KGL&lang=da&search_scope=MyInst_and_CI&adaptor=Primo%20Central&tab=Everything&query=any%2Ccontains%2CTricuspid%20annular%20plane%20systolic%20excursion%20is%20reduced%20in%20infants%20with%20pulmonary%20hypertension%3A%20value%20of%20tricuspid%20annular%20plane%20systolic%20excursion%20(TAPSE)%20to%20determine%20right%20ventricular%20function%20in%20various%20conditions%20of%20pediatric%20pulmonary%20hypertension).

^a^ Due to skewness of some of some outcome variables, transformations were necessary to obtain a normal distribution.

^b^ Linear scale. Adjusted mean differences on a linear scale; eg. 0.93 translates to a reduction of 7%

^c^ Boxcox-transformed to the power of 0.47

^d^ Boxcox-transformed to the power of 0.37

Reference group: infants born appropriate for gestational age (echocardiography: n=20,468, electrocardiography: n=14,076) [birthweight ≥10^th^ and <90^th^ percentile]).

All estimates are adjusted for newborn age at the time of cardiac examination, weight, length, and gestational age at birth, and sex.

eTable 6. Comparison of echocardiographic left ventricular measures and electrocardiographic measurements in infants born with fetal growth restriction or small for gestational age compared with infants born appropriate for gestational age.

Sensitivity analyses with exclusion of newborns with congenital heart defects

|  | Infants born with Fetal Growth Restriction (n=851) | | | Infants born Small for Gestational Age (n=1,955) | | |
| --- | --- | --- | --- | --- | --- | --- |
| Variable | Mean adjusted difference (95% CI) | | p-value | Mean adjusted difference (95% CI) | | p-value |
| E/A ratio | 0.01 | (-0.02, 0.03) | 0.46 | -0.01 | (-0.02, 0.01) | 0.54 |
| Stroke Volume (mL) | -0.02 | (-0.16, 0.11) | 0.74 | -0.09 | (-0.18, 0.00) | 0.06 |
| Fractional Shortening (%) | -0.01 | (-0.33, 0.32) | 0.97 | 0.11 | (-0.11, 0.32) | 0.34 |
| Ejection Fraction (%) | -0.01 | (-0.45, 0.44) | 0.97 | 0.15 | (-0.14, 0.44) | 0.32 |
| Mitral valve early peak velocity (cm/s) | 1.04 | (0.07, 2.02) | 0.04 | 0.96 | (0.30, 1.61) | <0.01 |
| Mitral valve atrial peak velocity (cm/s) | 0.49 | (-0.49, 1.47) | 0.33 | 1.09 | (0.43, 1.75) | <0.01 |
| Mitral valve deceleration time (ms) | 0.22 | (0.00, 0.44) | 0.05 | -0.05 | (-0.20, 0.10) | 0.52 |
| Left ventricular posterior wall end-diastolic thickness (mm) | -0.04 | (-0.08, 0.00) | 0.04 | -0.04 | (-0.07, -0.01) | <0.01 |
| Left ventricular internal diameter end-diastolic thickness (mm) | -0.13 | (-0.25, -0.02) | 0.03 | -0.14 | (-0.22, -0.06) | <0.001 |
| Left ventricular internal diameter end-systolic thickness(mm) | -0.09 | (-0.19, 0.00) | 0.06 | -0.11 | (-0.18, -0.05) | <0.001 |
| Interventricular septum end-diastole thickness (mm) | -0.07 | (-0.1, -0.03) | <0.001 | -0.03 | (-0.06, -0.01) | 0.01 |
| End-systolic volume (mL) | 0.00 | (-0.09, 0.09) | 0.97 | -0.06 | (-0.12, 0.00) | 0.04 |
| End-diastolic volume (mL) | -0.04 | (-0.23, 0.15) | 0.69 | -0.15 | (-0.27, -0.02) | 0.02 |
| TAPSE (mm) | -0.23 | (-0.35, -0.11) | <0.001 | -0.03 | (-0.11, 0.04) | 0.41 |
|  |  |  |  |  |  |  |
| Electrocardiographic parameters | Infants born with Fetal Growth Restriction (n=494) | | | Infants born Small for Gestational Age (n=1,269) | | |
| Heart rate (bpm) | 2.39 | (0.33, 4.44) | 0.02 | 2.52 | (1.19, 3.84) | <0.001 |
| QT interval, uncorrected (ms) | -3.79 | (-6.32, -1.26) | <0.01 | -1.37 | (-3.02, 0.27) | 0.10 |
| QTc Fridericia (ms) | -2.62 | (-4.88, -0.35) | 0.02 | 0.34 | (-1.14, 1.81) | 0.65 |
| QTc Bazett (ms) | -1.57 | (-4.02, 0.89) | 0.21 | 1.65 | (0.05, 3.25) | 0.04 |
| QRS duration (ms) | -0.46 | (-1.05, 0.13) | 0.13 | -0.39 | (-0.77, 0.00) | 0.05 |
| PR interval (ms) | 0.66 | (-0.48, 1.80) | 0.25 | 0.04 | (-0.69, 0.78) | 0.91 |
| QRS axis ^b^ | 1.01 | (0.98, 1.03) | 0.65 | 1.00 | (0.98, 1.01) | 0.53 |
| Max R-wave amplitude in V1 ^b^ | 0.99 | (0.94, 1.05) | 0.82 | 1.00 | (0.96, 1.03) | 0.85 |
| Max R-wave amplitude in V6 ^c^ | -0.59 | (-1.31, 0.13) | 0.11 | -0.46 | (-0.93, 0.01) | 0.05 |
| Max S-wave amplitude in V1 ^d^ | 0.34 | (0.02, 0.67) | 0.04 | 0.27 | (0.06, 0.48) | 0.01 |
| Max S-wave amplitude in V6 ^b^ | 0.95 | (0.89, 1.03) | 0.22 | 0.94 | (0.90, 0.99) | 0.01 |

Bpm; beats per minute; CI; confidence interval, ms; milliseconds, TAPSE; [Tricuspid Annular Plane Systolic Excursion](https://soeg.kb.dk/discovery/fulldisplay?docid=cdi_proquest_miscellaneous_1675876912&context=PC&vid=45KBDK_KGL:KGL&lang=da&search_scope=MyInst_and_CI&adaptor=Primo%20Central&tab=Everything&query=any%2Ccontains%2CTricuspid%20annular%20plane%20systolic%20excursion%20is%20reduced%20in%20infants%20with%20pulmonary%20hypertension%3A%20value%20of%20tricuspid%20annular%20plane%20systolic%20excursion%20(TAPSE)%20to%20determine%20right%20ventricular%20function%20in%20various%20conditions%20of%20pediatric%20pulmonary%20hypertension).

^a^ Due to skewness of some of some outcome variables, transformations were necessary to obtain a normal distribution.

^b^ Linear scale. Adjusted mean differences on a linear scale; eg. 0.93 translates to a reduction of 7%

^c^ Boxcox-transformed to the power of 0.47

^d^ Boxcox-transformed to the power of 0.37

Reference group: infants born appropriate for gestational age (echocardiography: n=19,078, electrocardiography: n=13,028) [birthweight ≥10^th^ and <90^th^ percentile]).

All estimates are adjusted for newborn age at the time of cardiac examination, weight, length, and gestational age at birth, and sex.

eTable 7. Comparison of echocardiographic left ventricular measures and electrocardiographic measurements in infants born with fetal growth restriction or small for gestational age compared with infants born appropriate for gestational age.

Sensitivity analyses with exclusion of premature born infants.

|  | Infants born with Fetal Growth Restriction (n=759) | | | Infants born Small for Gestational Age (n=1,936) | | |
| --- | --- | --- | --- | --- | --- | --- |
| Variable | Mean adjusted difference (95% CI) | | p-value | Mean adjusted difference (95% CI) | | p-value |
| E/A ratio | 0.01 | (-0.02, 0.03) | 0.55 | 0.00 | (-0.02, 0.01) | 0.55 |
| Stroke Volume (mL) | -0.02 | (-0.17, 0.12) | 0.73 | -0.07 | (-0.16, 0.02) | 0.12 |
| Fractional Shortening (%) | -0.16 | (-0.50, 0.18) | 0.35 | 0.15 | (-0.07, 0.36) | 0.18 |
| Ejection Fraction (%) | -0.21 | (-0.67, 0.25) | 0.37 | 0.21 | (-0.09, 0.50) | 0.17 |
| Mitral valve early peak velocity (cm/s) | 1.35 | (0.32, 2.37) | 0.01 | 0.97 | (0.31, 1.64) | <0.01 |
| Mitral valve atrial peak velocity (cm/s) | 1.01 | (-0.01, 2.03) | 0.05 | 1.15 | (0.49, 1.81) | <0.001 |
| Mitral valve deceleration time (ms) | 0.22 | (-0.01, 0.46) | 0.06 | -0.05 | (-0.20, 0.10) | 0.49 |
| Left ventricular posterior wall end-diastolic thickness (mm) | -0.03 | (-0.07, 0.01) | 0.13 | -0.03 | (-0.06, -0.01) | 0.01 |
| Left ventricular internal diameter end-diastolic thickness (mm) | -0.11 | (-0.23, 0.01) | 0.08 | -0.13 | (-0.21, -0.05) | <0.01 |
| Left ventricular internal diameter end-systolic thickness(mm) | -0.05 | (-0.15, 0.05) | 0.32 | -0.11 | (-0.18, -0.05) | <0.001 |
| Interventricular septum end-diastole thickness (mm) | -0.06 | (-0.10, -0.03) | <0.01 | -0.03 | (-0.06, -0.01) | 0.01 |
| End-systolic volume (mL) | 0.03 | (-0.07, 0.12) | 0.59 | -0.07 | (-0.13, -0.01) | 0.03 |
| End-diastolic volume (mL) | -0.02 | (-0.22, 0.18) | 0.83 | -0.14 | (-0.26, -0.01) | 0.04 |
| TAPSE (mm) | -0.15 | (-0.27, -0.03) | 0.01 | -0.03 | (-0.11, 0.04) | 0.39 |
|  |  |  |  |  |  |  |
| Electrocardiographic parameters | Infants born with Fetal Growth Restriction (n=491) | | | Infants born Small for Gestational Age (n=1,308) | | |
| Heart rate (bpm) | 2.14 | (0.08, 4.20) | 0.04 | 2.68 | (1.37, 3.99) | <0.001 |
| QT interval, uncorrected (ms) | -3.03 | (-5.58, -0.48) | 0.02 | -1.54 | (-3.17, 0.09) | 0.06 |
| QTc Fridericia (ms) | -1.90 | (-4.18, 0.39) | 0.10 | 0.24 | (-1.22, 1.70) | 0.75 |
| QTc Bazett (ms) | -0.91 | (-3.38, 1.56) | 0.47 | 1.59 | (0.01, 3.17) | 0.05 |
| QRS duration (ms) | -0.43 | (-1.02, 0.16) | 0.15 | -0.37 | (-0.75, 0.00) | 0.05 |
| PR interval (ms) | 0.87 | (-0.27, 2.01) | 0.13 | 0.22 | (-0.50, 0.95) | 0.55 |
| QRS axis ^b^ | 1.00 | (0.98, 1.03) | 0.76 | 1.00 | (0.98, 1.01) | 0.69 |
| Max R-wave amplitude in V1 ^b^ | 1.00 | (0.95, 1.05) | 0.97 | 0.99 | (0.96, 1.02) | 0.55 |
| Max R-wave amplitude in V6 ^c^ | -0.80 | (-1.52, -0.08) | 0.03 | -0.62 | (-1.08, -0.16) | 0.01 |
| Max S-wave amplitude in V1 ^d^ | 0.23 | (-0.10, 0.55) | 0.17 | 0.22 | (0.01, 0.43) | 0.04 |
| Max S-wave amplitude in V6 ^b^ | 0.94 | (0.88, 1.02) | 0.15 | 0.93 | (0.89, 0.97) | <0.01 |
|  |  |  |  |  |  |  |

Bpm; beats per minute; CI; confidence interval, ms; milliseconds, TAPSE; [Tricuspid Annular Plane Systolic Excursion](https://soeg.kb.dk/discovery/fulldisplay?docid=cdi_proquest_miscellaneous_1675876912&context=PC&vid=45KBDK_KGL:KGL&lang=da&search_scope=MyInst_and_CI&adaptor=Primo%20Central&tab=Everything&query=any%2Ccontains%2CTricuspid%20annular%20plane%20systolic%20excursion%20is%20reduced%20in%20infants%20with%20pulmonary%20hypertension%3A%20value%20of%20tricuspid%20annular%20plane%20systolic%20excursion%20(TAPSE)%20to%20determine%20right%20ventricular%20function%20in%20various%20conditions%20of%20pediatric%20pulmonary%20hypertension).

^a^ Due to skewness of some of some outcome variables, transformations were necessary to obtain a normal distribution.

^b^ Linear scale. Adjusted mean differences on a linear scale; eg. 0.93 translates to a reduction of 7%

^c^ Boxcox-transformed to the power of 0.47

^d^ Boxcox-transformed to the power of 0.37

Reference group: infants born appropriate for gestational age (echocardiography: n=19,610, electrocardiography: n=13,624) [birthweight ≥10^th^ and <90^th^ percentile]).

All estimates are adjusted for newborn age at the time of cardiac examination, weight, length, and gestational age at birth, and sex.

eTable 8. Comparison of echocardiographic left ventricular measures and electrocardiographic measurements in infants born with fetal growth restriction or small for gestational age compared with infants born appropriate for gestational age.

Sensitivity analyses with exclusion of infants born to mothers with pre-existing diabetes.

|  | Infants born with Fetal Growth Restriction (n=926) | | | Infants born Small for Gestational Age (n=2,099) | | |
| --- | --- | --- | --- | --- | --- | --- |
| Variable | Mean adjusted difference (95% CI) | | p-value | Mean adjusted difference (95% CI) | | p-value |
| E/A ratio | 0.01 | (-0.02, 0.03) | 0.50 | 0.00 | (-0.02, 0.01) | 0.65 |
| Stroke Volume (mL) | -0.02 | (-0.15, 0.11) | 0.80 | -0.08 | (-0.17, 0.00) | 0.06 |
| Fractional Shortening (%) | -0.03 | (-0.35, 0.28) | 0.84 | 0.14 | (-0.07, 0.35) | 0.18 |
| Ejection Fraction (%) | -0.03 | (-0.46, 0.40) | 0.88 | 0.21 | (-0.08, 0.49) | 0.15 |
| Mitral valve early peak velocity (cm/s) | 1.00 | (0.05, 1.95) | 0.04 | 0.97 | (0.33, 1.61) | <0.01 |
| Mitral valve atrial peak velocity (cm/s) | 0.54 | (-0.41, 1.49) | 0.26 | 0.99 | (0.35, 1.64) | <0.01 |
| Mitral valve deceleration time (ms) | 0.20 | (-0.02, 0.42) | 0.07 | -0.04 | (-0.18, 0.11) | 0.61 |
| Left ventricular posterior wall end-diastolic thickness (mm) | -0.05 | (-0.09, -0.01) | 0.01 | -0.04 | (-0.07, -0.01) | <0.01 |
| Left ventricular internal diameter end-diastolic thickness (mm) | -0.12 | (-0.24, -0.01) | 0.03 | -0.14 | (-0.22, -0.07) | <0.001 |
| Left ventricular internal diameter end-systolic thickness(mm) | -0.09 | (-0.18, 0.01) | 0.07 | -0.12 | (-0.18, -0.06) | <0.001 |
| Interventricular septum end-diastole thickness (mm) | -0.07 | (-0.11, -0.04) | <0.001 | -0.03 | (-0.06, -0.01) | 0.01 |
| End-systolic volume (mL) | 0.00 | (-0.09, 0.08) | 0.98 | -0.07 | (-0.13, -0.02) | 0.01 |
| End-diastolic volume (mL) | -0.03 | (-0.21, 0.16) | 0.77 | -0.16 | (-0.28, -0.03) | 0.01 |
| TAPSE (mm) | -0.20 | (-0.32, -0.09) | <0.001 | -0.04 | (-0.12, 0.03) | 0.29 |
|  |  |  |  |  |  |  |
| Electrocardiographic parameters | Infants born with Fetal Growth Restriction (n=544) | | | Infants born Small for Gestational Age (n=1,380) | | |
| Heart rate (bpm) | 2.38 | (0.42, 4.34) | 0.02 | 2.50 | (1.23, 3.78) | <0.001 |
| QT interval, uncorrected (ms) | -3.20 | (-5.62, -0.78) | 0.01 | -1.60 | (-3.18, -0.02) | 0.05 |
| QTc Fridericia (ms) | -2.13 | (-4.30, 0.05) | 0.06 | 0.05 | (-1.37, 1.47) | 0.94 |
| QTc Bazett (ms) | -1.16 | (-3.52, 1.19) | 0.33 | 1.33 | (-0.21, 2.87) | 0.09 |
| QRS duration (ms) | -0.42 | (-0.98, 0.15) | 0.15 | -0.36 | (-0.73, 0.01) | 0.06 |
| PR interval (ms) | 0.84 | (-0.25, 1.92) | 0.13 | 0.11 | (-0.59, 0.82) | 0.75 |
| QRS axis ^b^ | 1.00 | (0.98, 1.03) | 0.70 | 1.00 | (0.98, 1.01) | 0.73 |
| Max R-wave amplitude in V1 ^b^ | 1.00 | (0.95, 1.05) | 0.89 | 0.99 | (0.96, 1.03) | 0.72 |
| Max R-wave amplitude in V6 ^c^ | -0.55 | (-1.23, 0.14) | 0.12 | -0.63 | (-1.07, -0.18) | 0.01 |
| Max S-wave amplitude in V1 ^d^ | 0.24 | (-0.07, 0.55) | 0.13 | 0.26 | (0.06, 0.46) | 0.01 |
| Max S-wave amplitude in V6 ^b^ | 0.96 | (0.90, 1.04) | 0.32 | 0.93 | (0.89, 0.98) | <0.01 |

Bpm; beats per minute; CI; confidence interval, ms; milliseconds, TAPSE; [Tricuspid Annular Plane Systolic Excursion](https://soeg.kb.dk/discovery/fulldisplay?docid=cdi_proquest_miscellaneous_1675876912&context=PC&vid=45KBDK_KGL:KGL&lang=da&search_scope=MyInst_and_CI&adaptor=Primo%20Central&tab=Everything&query=any%2Ccontains%2CTricuspid%20annular%20plane%20systolic%20excursion%20is%20reduced%20in%20infants%20with%20pulmonary%20hypertension%3A%20value%20of%20tricuspid%20annular%20plane%20systolic%20excursion%20(TAPSE)%20to%20determine%20right%20ventricular%20function%20in%20various%20conditions%20of%20pediatric%20pulmonary%20hypertension).

^a^ Due to skewness of some of some outcome variables, transformations were necessary to obtain a normal distribution.

^b^ Linear scale. Adjusted mean differences on a linear scale; eg. 0.93 translates to a reduction of 7%

^c^ Boxcox-transformed to the power of 0.47

^d^ Boxcox-transformed to the power of 0.37

Reference group: infants born appropriate for gestational age (echocardiography: n=20,350, electrocardiography: n=14,007) [birthweight ≥10^th^ and <90^th^ percentile]).

All estimates are adjusted for newborn age at the time of cardiac examination, weight, length, and gestational age at birth, and sex.

eTable 9. Comparison of echocardiographic left ventricular measures and electrocardiographic measurements in infants born with fetal growth restriction or small for gestational age compared with infants born appropriate for gestational age.

Sensitivity analyses with exclusion of infants born to mothers with congenital heart defects.

|  | Infants born with Fetal Growth Restriction (n=924) | | | Infants born Small for Gestational Age (n=2,102) | | |
| --- | --- | --- | --- | --- | --- | --- |
| Variable | Mean adjusted difference (95% CI) | | p-value | Mean adjusted difference (95% CI) | | p-value |
| E/A ratio | 0.01 | (-0.02, 0.03) | 0.51 | 0.00 | (-0.02, 0.01) | 0.66 |
| Stroke Volume (mL) | -0.02 | (-0.15, 0.12) | 0.82 | -0.08 | (-0.17, 0.00) | 0.06 |
| Fractional Shortening (%) | -0.03 | (-0.34, 0.29) | 0.87 | 0.16 | (-0.05, 0.37) | 0.13 |
| Ejection Fraction (%) | -0.03 | (-0.45, 0.4) | 0.91 | 0.23 | (-0.06, 0.52) | 0.11 |
| Mitral valve early peak velocity (cm/s) | 0.94 | (-0.01, 1.89) | 0.05 | 0.95 | (0.31, 1.59) | <0.01 |
| Mitral valve atrial peak velocity (cm/s) | 0.51 | (-0.44, 1.46) | 0.30 | 0.98 | (0.34, 1.62) | <0.01 |
| Mitral valve deceleration time (ms) | 0.21 | (-0.01, 0.42) | 0.06 | -0.03 | (-0.18, 0.11) | 0.68 |
| Left ventricular posterior wall end-diastolic thickness (mm) | -0.05 | (-0.09, -0.01) | 0.01 | -0.04 | (-0.07, -0.02) | <0.01 |
| Left ventricular internal diameter end-diastolic thickness (mm) | -0.12 | (-0.24, -0.01) | 0.03 | -0.15 | (-0.22, -0.07) | <0.001 |
| Left ventricular internal diameter end-systolic thickness(mm) | -0.09 | (-0.18, 0.01) | 0.07 | -0.13 | (-0.19, -0.06) | <0.001 |
| Interventricular septum end-diastole thickness (mm) | -0.07 | (-0.11, -0.04) | <0.001 | -0.03 | (-0.06, -0.01) | 0.01 |
| End-systolic volume (mL) | 0.00 | (-0.09, 0.08) | 0.98 | -0.08 | (-0.13, -0.02) | 0.01 |
| End-diastolic volume (mL) | -0.03 | (-0.21, 0.16) | 0.79 | -0.16 | (-0.28, -0.04) | 0.01 |
| TAPSE (mm) | -0.20 | (-0.31, -0.09) | <0.001 | -0.04 | (-0.12, 0.03) | 0.29 |
|  |  |  |  |  |  |  |
| Electrocardiographic parameters | Infants born with Fetal Growth Restriction (n=544) | | | Infants born Small for Gestational Age (n=1,382) | | |
| Heart rate (bpm) | 2.38 | (0.42, 4.34) | 0.02 | 2.48 | (1.2, 3.75) | <0.001 |
| QT interval, uncorrected (ms) | -3.23 | (-5.65, -0.81) | 0.01 | -1.61 | (-3.19, -0.03) | 0.05 |
| QTc Fridericia (ms) | -2.18 | (-4.35, 0.00) | 0.05 | 0.02 | (-1.4, 1.44) | 0.98 |
| QTc Bazett (ms) | -1.22 | (-3.58, 1.13) | 0.31 | 1.28 | (-0.26, 2.82) | 0.10 |
| QRS duration (ms) | -0.41 | (-0.98, 0.16) | 0.15 | -0.36 | (-0.73, 0.01) | 0.06 |
| PR interval (ms) | 0.88 | (-0.21, 1.96) | 0.11 | 0.10 | (-0.6, 0.81) | 0.77 |
| QRS axis ^b^ | 1.00 | (0.98, 1.03) | 0.69 | 1.00 | (0.98, 1.01) | 0.74 |
| Max R-wave amplitude in V1 ^b^ | 1.00 | (0.95, 1.04) | 0.85 | 0.99 | (0.96, 1.02) | 0.66 |
| Max R-wave amplitude in V6 ^c^ | -0.56 | (-1.25, 0.12) | 0.11 | -0.62 | (-1.07, -0.17) | 0.01 |
| Max S-wave amplitude in V1 ^d^ | 0.24 | (-0.07, 0.55) | 0.14 | 0.26 | (0.06, 0.46) | 0.01 |
| Max S-wave amplitude in V6 ^b^ | 0.96 | (0.90, 1.04) | 0.34 | 0.93 | (0.89, 0.98) | <0.01 |

Bpm; beats per minute; CI; confidence interval, ms; milliseconds, TAPSE; [Tricuspid Annular Plane Systolic Excursion](https://soeg.kb.dk/discovery/fulldisplay?docid=cdi_proquest_miscellaneous_1675876912&context=PC&vid=45KBDK_KGL:KGL&lang=da&search_scope=MyInst_and_CI&adaptor=Primo%20Central&tab=Everything&query=any%2Ccontains%2CTricuspid%20annular%20plane%20systolic%20excursion%20is%20reduced%20in%20infants%20with%20pulmonary%20hypertension%3A%20value%20of%20tricuspid%20annular%20plane%20systolic%20excursion%20(TAPSE)%20to%20determine%20right%20ventricular%20function%20in%20various%20conditions%20of%20pediatric%20pulmonary%20hypertension).

^a^ Due to skewness of some of some outcome variables, transformations were necessary to obtain a normal distribution.

^b^ Linear scale. Adjusted mean differences on a linear scale; eg. 0.93 translates to a reduction of 7%

^c^ Boxcox-transformed to the power of 0.47

^d^ Boxcox-transformed to the power of 0.37

Reference group: infants born appropriate for gestational age (echocardiography: n=20,434, electrocardiography: n=14,052) [birthweight ≥10^th^ and <90^th^ percentile]).

All estimates are adjusted for newborn age at the time of cardiac examination, weight, length, and gestational age at birth, and sex.

eTable 10. Comparison of echocardiographic left ventricular measures and electrocardiographic measurements in infants born with fetal growth restriction or small for gestational age compared with infants born appropriate for gestational age.

Sensitivity analyses with exclusion of newborns exposed to preeclampsia.

|  | Infants born with Fetal Growth Restriction (n=841) | | | Infants born Small for Gestational Age (n=2,019) | | |
| --- | --- | --- | --- | --- | --- | --- |
| Variable | Mean adjusted difference (95% CI) | | p-value | Mean adjusted difference (95% CI) | | p-value |
| E/A ratio | 0.01 | (-0.01, 0.03) | 0.44 | -0.01 | (-0.02, 0.01) | 0.51 |
| Stroke Volume (mL) | -0.01 | (-0.15, 0.12) | 0.86 | -0.09 | (-0.18, 0.00) | 0.06 |
| Fractional Shortening (%) | -0.03 | (-0.36, 0.29) | 0.85 | 0.13 | (-0.08, 0.34) | 0.24 |
| Ejection Fraction (%) | -0.03 | (-0.48, 0.41) | 0.89 | 0.19 | (-0.11, 0.48) | 0.21 |
| Mitral valve early peak velocity (cm/s) | 1.10 | (0.11, 2.08) | 0.03 | 0.81 | (0.16, 1.46) | 0.02 |
| Mitral valve atrial peak velocity (cm/s) | 0.61 | (-0.37, 1.6) | 0.22 | 0.97 | (0.32, 1.62) | <0.01 |
| Mitral valve deceleration time (ms) | 0.25 | (0.03, 0.48) | 0.03 | -0.03 | (-0.18, 0.12) | 0.71 |
| Left ventricular posterior wall end-diastolic thickness (mm) | -0.05 | (-0.09, -0.01) | 0.02 | -0.04 | (-0.06, -0.01) | <0.01 |
| Left ventricular internal diameter end-diastolic thickness (mm) | -0.13 | (-0.24, -0.01) | 0.03 | -0.15 | (-0.22, -0.07) | <0.001 |
| Left ventricular internal diameter end-systolic thickness(mm) | -0.08 | (-0.18, 0.01) | 0.09 | -0.12 | (-0.18, -0.06) | <0.001 |
| Interventricular septum end-diastole thickness (mm) | -0.08 | (-0.11, -0.04) | <0.001 | -0.03 | (-0.06, -0.01) | 0.01 |
| End-systolic volume (mL) | 0.00 | (-0.09, 0.09) | 0.97 | -0.07 | (-0.13, -0.01) | 0.01 |
| End-diastolic volume (mL) | -0.04 | (-0.23, 0.15) | 0.69 | -0.16 | (-0.28, -0.03) | 0.01 |
| TAPSE (mm) | -0.17 | (-0.29, -0.07) | <0.01 | -0.04 | (-0.12, 0.03) | 0.27 |
|  |  | | |  | | |
| Electrocardiographic parameters | Infants born with Fetal Growth Restriction (n=503) | | | Infants born Small for Gestational Age (n=1,336) | | |
| Heart rate (bpm) | 1.91 | (-0.13, 3.94) | 0.07 | 2.43 | (1.14, 3.73) | <0.001 |
| QT interval, uncorrected (ms) | -2.83 | (-5.33, -0.32) | 0.03 | -1.42 | (-3.03, 0.19) | 0.08 |
| QTc Fridericia (ms) | -1.86 | (-4.10, 0.39) | 0.11 | 0.25 | (-1.19, 1.70) | 0.73 |
| QTc Bazett (ms) | -0.98 | (-3.41, 1.45) | 0.43 | 1.55 | (-0.01, 3.11) | 0.05 |
| QRS duration (ms) | -0.27 | (-0.85, 0.32) | 0.37 | -0.38 | (-0.76, -0.01) | 0.04 |
| PR interval (ms) | 1.00 | (-0.13, 2.12) | 0.08 | 0.13 | (-0.58, 0.85) | 0.71 |
| QRS axis ^b^ | 1.00 | (0.98, 1.03) | 0.74 | 1.00 | (0.98, 1.01) | 0.68 |
| Max R-wave amplitude in V1 ^b^ | 1.00 | (0.95, 1.05) | 0.97 | 0.99 | (0.96, 1.03) | 0.75 |
| Max R-wave amplitude in V6 ^c^ | -0.60 | (-1.31, 0.11) | 0.10 | -0.62 | (-1.08, -0.17) | 0.01 |
| Max S-wave amplitude in V1 ^d^ | 0.31 | (-0.01, 0.63) | 0.06 | 0.28 | (0.07, 0.48) | 0.01 |
| Max S-wave amplitude in V6 ^b^ | 0.96 | (0.90, 1.03) | 0.29 | 0.92 | (0.88, 0.97) | <0.001 |

Bpm; beats per minute; CI; confidence interval, ms; milliseconds, TAPSE; [Tricuspid Annular Plane Systolic Excursion](https://soeg.kb.dk/discovery/fulldisplay?docid=cdi_proquest_miscellaneous_1675876912&context=PC&vid=45KBDK_KGL:KGL&lang=da&search_scope=MyInst_and_CI&adaptor=Primo%20Central&tab=Everything&query=any%2Ccontains%2CTricuspid%20annular%20plane%20systolic%20excursion%20is%20reduced%20in%20infants%20with%20pulmonary%20hypertension%3A%20value%20of%20tricuspid%20annular%20plane%20systolic%20excursion%20(TAPSE)%20to%20determine%20right%20ventricular%20function%20in%20various%20conditions%20of%20pediatric%20pulmonary%20hypertension).

^a^ Due to skewness of some of some outcome variables, transformations were necessary to obtain a normal distribution.

^b^ Linear scale. Adjusted mean differences on a linear scale; eg. 0.93 translates to a reduction of 7%

^c^ Boxcox-transformed to the power of 0.47

^d^ Boxcox-transformed to the power of 0.37

Reference group: infants born appropriate for gestational age (echocardiography: n=19,951, electrocardiography: n=13,765) [birthweight ≥10^th^ and <90^th^ percentile]).

All estimates are adjusted for newborn age at the time of cardiac examination, weight, length, and gestational age at birth, and sex.

**Supplementary material**

Their findings have given us the following two polynomial regression equations to calculate the estimated body weight:

$$Boys:f\left( x \right)=-1.907345*{10}^{-6}*x^{4}+1.140644*{10}^{-3}*x^{3}-1.336265*{10}^{-1}*x^{2}+1.976961*x+2.410053*{10}^{2}$$

$$Girls:f\left( x \right)= -2.761948*{10}^{-6}*x^{4}+1.744841*{10}^{-3}*x^{3}-2.893626*{10}^{-1}*x^{2}+1.891197*{10}^{1}*x-4.135122*{10}^{2}$$

Where f(x) = estimated fetal weight and x = gestational age in days. These formulas were calculated on Scandinavian newborns, like the ones included in this study. K. Maršál et. Al found a standard deviation of 12%. The FGR, SGA and large for gestational age cutoffs have been calculated for each using the inverse transformation formula:

$$X=Z\cdot\sigma+\mu$$

Where X = the value corresponding to the percentile (3^rd^, 10^th^ and 90^th^), Z = Z-score and the corresponding Z-scores of the 3^rd^, 10^th^ and 90^th^ percentiles, which are -1.880794, -1.281552 and 1.281552, σ = standard deviation and μ = 0 as we have a normal distribution

By calculating the following we find the expected weight at a certain percentile at a certain gestational age for each child.

$$X=f\left( x \right)+f\left( x \right)*Z\cdot\sigma$$

By comparing their actual birth weight with the expected birth weights at the 3^rd^, 10^th^ and 90^th^ percentile we have divide the newborns into the groups: FGR, SGA, appropriate for gestational age and large for gestational age.
